# From Theory to Experiment: Reviewing the Role of Graphene in Li-Ion Batteries Through Density Functional Theory

**DOI:** 10.3390/nano15130992

**Published:** 2025-06-26

**Authors:** Ghada AlJaber, Basheer AlShammari, Bandar AlOtaibi

**Affiliations:** 1 Advanced Material Technology Institute, King Abdelaziz City for Science and Technology, Riyadh 11442, Saudi Arabia; 437200330@student.ksu.edu.sa (G.A.);; 2 Ministry of Environment, Water and Agriculture, Riyadh 11195, Saudi Arabia

**Keywords:** energy storage materials, graphene, lithium-ion batteries, density functional theory

## Abstract

Rechargeable Lithium-ion batteries (LIBs) have experienced swift advancement and widespread commercialization in electronic devices and electric vehicles, driven by their exceptional efficiency, energy capacity, and elevated power density. However, to promote sustainable energy development there is a dire need to further extend the search for developing and optimizing the existing anode active energy storage materials. This has steered research towards carbon-based anode materials, particularly graphene, to promote and develop sustainable and efficient LIB technology that can drive the next wave of industrial innovation. In this regard, density functional theory (DFT) computations are considered a powerful tool to elucidate chemical and physical properties at an atomistic scale and serve as a transformative framework, catalyzing the discovery of novel high-performance anode materials for LIBs. This review highlights the computational progress in graphene and graphene composites to design better graphene-based anode materials for LIBs.

## 1. Introduction

Lithium-ion batteries (LIBs) are essential power sources for portable electronics and electric vehicles (EVs). They outperform other secondary batteries like Nickel–Cadmium (NiCd) and Nickel–Metal Hydride (NiMH) due to higher energy density, lightweight design, long cycle life, and minimal memory effect [1,2]. These advantages make LIBs dominant in rechargeable systems, with potential in large-scale renewable energy infrastructure [3,4]. However, scaling LIBs for large applications requires improvements in energy density, power density, lifespan, cost-effectiveness, and safety. Addressing this requires a deeper understanding of electrochemical behavior, structural intricacies, and energy storage strategies. The push for environmental sustainability and carbon (C)-neutral energy solutions has accelerated the transition to clean energy [5]. Currently, most LIBs use graphite as an anode material, limiting capacity to 372 mAh g⁻^1^ [6], spurring research into alternative materials with better surface areas and improved volumetric expansion.

Graphene, a monolayer of sp^2^-bonded C atoms in a two-dimensional (2D) honeycomb lattice, is emerging as a key material for high-performance LIB anodes. Its structural and electronic properties set it apart from graphite, with a high theoretical surface area (~2630 m^2^/g) and inherent conductivity, enabling ultrafast charge–discharge cycles by minimizing electron and ion transport resistances [7]. The atomic thickness of graphene eliminates bulk diffusion barriers for lithium-ions, promoting intercalation kinetics and rapid ionic conduction. This combined with mechanical robustness allows graphene to accommodate large volumetric changes during repeated lithiation/delithiation cycles, extending battery lifespan [8,9]. Additionally, graphene’s versatility enables hybrid structures with high-capacity anode materials, enhancing both energy storage and cyclic stability. Its high surface area provides numerous active sites for lithium-ion adsorption, improving capacity beyond conventional graphite [5,10]. These properties make graphene ideal for high-energy-density, sustainable electrodes. Traditional exploration of electrode materials has followed a structure–property approach, which, though insightful, is labor-intensive. Computational methods, particularly density functional theory (DFT), have revolutionized materials research by enabling predictive modeling of material properties at atomic scales [11]. DFT calculates ground state energies as a function of electron density, encompassing terms for non-interacting electrons, nuclei potentials, Coulombic interactions, and exchange correlation. For instance, Yildirim et al. [12] studied lithium (Li) adsorption on graphene with defects, concluding that defect density enhances capacity. Lohavi et al. [13] investigated graphene anodes doped with silicon (Si) and germanium (Ge), improving electrical properties. Wang et al. [14] used DFT to study how combining pentagonal graphene with Molybdenum disulfide (MoS_2_) enhances stability in LIBs. Hybrid functionals like the Perdew–Burke–Ernzerhof hybrid (PBE0) and Heyd–Scuseria–Ernzerhof (HSE06) improve accuracy in atomic and electronic structures [15]. Additionally, Hubbard U correction (U) values address electron localization issues, especially in transition metals and rare earth elements in LIB electrode materials [16]. DFT, combining a plane wave basis set with pseudopotentials, simplifies computation by focusing on valence electrons, enabling dynamic simulations [17]. Coupling DFT with Monte Carlo or molecular dynamics extends its predictive scope, making it essential for studying energy-related materials, particularly LIB electrodes [18,19,20].

Despite progress, there is a gap in reviews integrating theoretical and experimental insights into graphene’s role as an anode material in LIBs. Many studies focus on either computational models or experimental findings in isolation, limiting a complete understanding of graphene’s potential. This review aims to bridge this gap by combining insights from both DFT studies and experimental data, offering a comprehensive analysis of graphene’s role in LIBs. Linking DFT predictions with empirical findings deepens our understanding of graphene’s electrochemical behavior, ion diffusion, and structural adaptability, which are crucial for improving LIB performance. It also highlights how combining graphene with other high-capacity materials in composite structures could enhance capacity, stability, and efficiency, addressing current LIB limitations.

## 2. Density Functional Theory in Lithium-Ion Batteries

**Figure 1:**
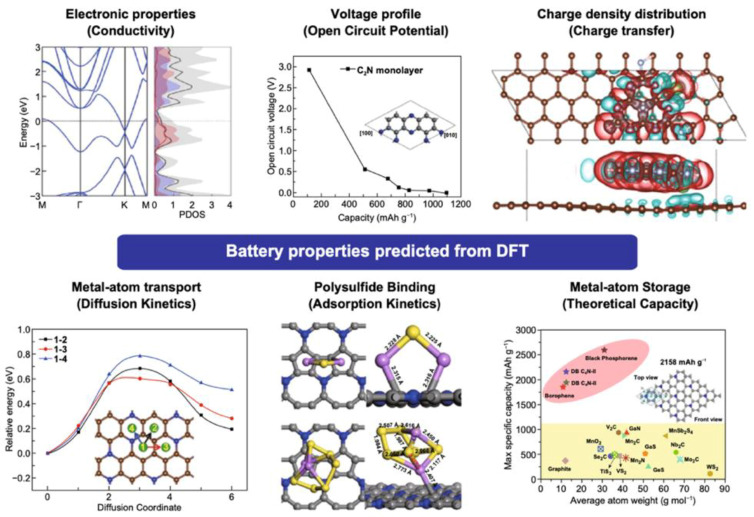
DFT calculations can be used to predict the electrochemical properties of batteries. Reproduced with permission [21]. Copyright 2021, Springer Nature.

DFT calculations offer a comprehensive approach for elucidating the intrinsic properties of materials and interaction mechanisms within the battery, which are important for the development of anode materials for LIBs. In recent decades, graphene has emerged as a promising anode for lithium batteries, yet a deep understanding of its operation within batteries remains challenging. Through DFT, we can predict several critical parameters that define electrode quality and performance, including theoretical capacitance, open circuit voltage, electronic properties, adsorption, diffusion, and charge transfer mechanisms (Figure 1). In the subsequent section, these aspects are discussed in detail, along with the challenges faced during DFT calculation.

### 2.1. Open Circuit Voltage and Theoretical Capacity

**Figure 2:**
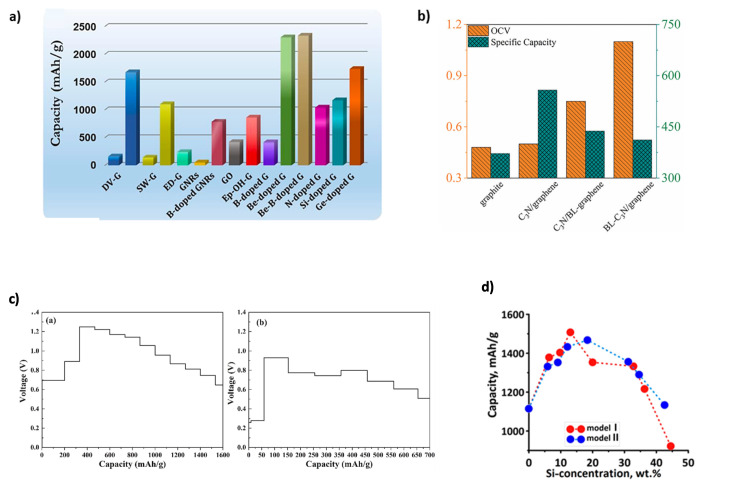
(**a**) Li storage capacity of graphene vs. graphene-based materials. Reproduced with permission [34]. Copyright 2021, Elsevier. (**b**) Interface effect on Li storage in C_3_N/graphene heterostructure. Reproduced with permission [27]. Copyright 2022, Elsevier. (**c**) OCV profiles of F-GDY and F-GDY/graphene. Adapted with permission [28]. Copyright 2022, Elsevier. (**d**) Porous 2D silicon-filled composite based on graphene and CNTs. Reproduced with permission [29]. Copyright 2020, MDPI.

The capacity of LIBs is intrinsically tied to the electrode material, which governs the maximum number of intercalated lithium ions (Li^+^). As shown in Figure 2a, graphene-based materials often demonstrate superior lithium storage capacity compared to traditional materials. The theoretical capacity is calculated using the following equation [22]:

$$C=\frac{nN_{A}e}{\varepsilon M}$$

where n is the number of intercalated ions, N_A_ is Avogadro’s number, e is the electric charge of the electron, ε is the conversion ratio of mAh to Coulomb (ε = 3.6), and M is the molecular weight of the substrate. Open-circuit voltage (OCV), reflecting the maximum electrochemical potential difference in the absence of current, is a critical factor in determining energy density. It serves as an intrinsic metric for energy storage potential, free from dynamic losses such as internal resistance [23,24]. OCV can be approximated from the change in internal energy, assuming negligible entropy and volume changes [23]:

$$V_{OC}=\frac{\Delta E}{\Delta xF}$$

where ΔE is the internal energy, F is Faraday’s constant, and Δx is the ion concentration [25]. Theoretically, the OCV can be obtained from the thermodynamic calculation of the electrode material [23]. Graphene possesses a theoretical capacity of 744 mAh g^−1^ [6], though its practical application faces challenges. Ullah et al. [26] applied DFT with the Spanish Initiative for Electronic Simulations with Thousands of Atoms (SIESTA) computational code to study beryllium (Be)-doped graphene, where Be induces electron deficiency, enhancing Li adsorption. They reported a significantly increased theoretical capacity of 2303.295 mAh g^−1^. The Li potential dropped with increasing concentration (1 to 12 atoms in single-vacancy and 1 to 16 atoms in vacancy-doped graphene), suggesting strong capacity retention and cycle stability. Gavali et al. [27] examined C_3_N (two-dimensional polyaniline)/graphene-based heterostructures via DFT (Quantum Espresso), showing that interfacial charge redistribution affects both OCV and specific capacity. For C_3_N/graphene, C_3_N/bilayer graphene, and bilayer C_3_N/graphene, OCVs ranged from 0.45–0.05 V, 0.48–0.05 V, to 1.05–0.08 V, respectively. Theoretical capacities were 558, 458, and 423 mAh g^−1^ at varying Li concentrations (x = 1.5, 1.3, and 1.2), indicating how structural configuration tunes electrochemical performance (Figure 2b). Kong et al. [28] found that strong fluorine (F)–Li interactions in fluorinated graphdiyne (F-GDY) lead to fluorine detachment and structural instability. In contrast, the F-GDY/graphene composite moderates this interaction, enhancing structural stability. As shown in Figure 2c, F-GDY shows a sharp OCV rise (~1.22 V at 400 mAh g^−1^), whereas the composite maintains OCV below 1.0 V across a wider capacity range. Theoretical capacities are 385.5, 1661.0, and 680.3 mAh g^−1^ for graphene, F-GDY, and the composite, respectively. Kolosov and Glukhova [29] used the SCC-DFTB method to simulate Si nanoclusters embedded in graphene pores supported on single-walled C nanotube bases (SWCNTs). Their approach, implemented via dftb+ and Kvazar–Mizar, revealed that a silicon mass fraction of ~13–18 wt% optimally balances stability and lithium uptake, leading to theoretical capacities of ~1500 mAh g^−1^ and lithium intercalation of 27% and 45% in different structural models (Figure 2d). As these examples show, doping and heterostructure engineering significantly improve both OCV and theoretical capacity. Mousavi-Khoshdel et al. [30] demonstrated that co-doping graphene with nitrogen (N) and Si induces synergistic structural and electronic changes near the Fermi level, enhancing quantum capacitance beyond that of singly doped systems.

However, these predictions are often based on idealized conditions and fail to reflect the complexities of real electrochemical environments. To better evaluate practical performance, some researchers calculate the working voltage, which considers internal resistance and current during cycling. For example, Lu et al. [31] used DFT to study cyclohexanehexone (C_6_O_6_) as a high-capacity LIB cathode, analyzing its electronic structure including the total density of states (TDOS) and the highest occupied molecular orbital–lowest unoccupied molecular orbital (HOMO–LUMO) gap and identifying lithium binding sites. Nonetheless, modeling working voltage with DFT remains challenging due to factors like the electrical double layer, electrolyte effects, and reaction kinetics, including discharge product decomposition [32,33]. These limitations highlight the gap between theoretical predictions and real-world performance, though advancements in multi-scale modeling and experimental validation are gradually bridging this divide.

### 2.2. Electronic Structure

**Figure 3:**
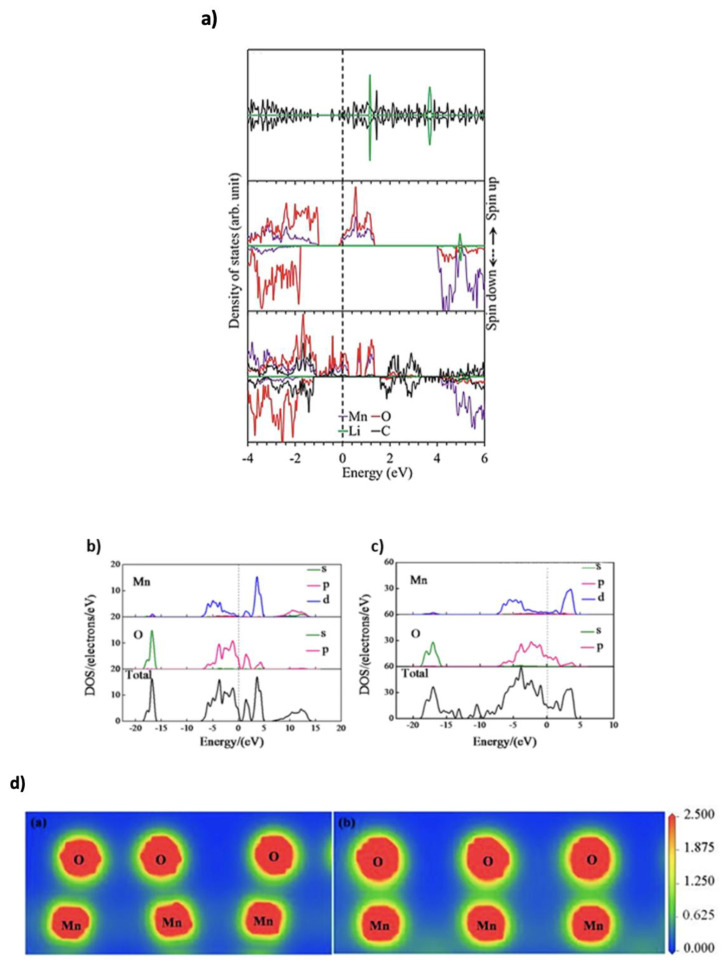
(**a**) DOS of Li adsorbed on graphene, MnO_2_, and MnO_2_/graphene. The colored lines represent the PDOS from different atomic species: Mn (purple), O (red), Li (green), and C (black). Reproduced with permission [42]. Copyright 2016, World Scientific. (**b**,**c**) DOS and PDOS of λ-MnO_2_ and λ-MnO_2_/graphene, respectively. PDOS from Mn and O atoms; s (green), p (pink), d (blue). (**d**) Electron density maps of O and Mn in λ-MnO_2_ and λ-MnO_2_/graphene. Reproduced with permission [43]. Copyright 2019, Royal Society of Chemistry.

The electronic structure of electrode materials, including their band structure and density of states (DOS), plays a pivotal role in LIB performance by influencing energy density, power delivery, and cycling stability. Graphene, known for its high electron mobility and Dirac cone structure, is especially promising for high-rate LIBs due to its exceptional electrical conductivity [35]. For advanced C-based materials such as doped graphene and graphene oxide (GO), DFT has proven instrumental in predicting how defect engineering, heteroatom doping, and surface functionalization alter their electronic properties. These modifications directly impact lithium-ion adsorption, charge transfer efficiency, and the Fermi level position, which are key to improving electrochemical kinetics and storage capacity. Li^+^ adsorption significantly modifies graphene’s electronic structure by introducing new states near the Fermi level. This is primarily due to charge transfer from Li^+^ to the graphene lattice, resulting in a shift in energy levels and often a reduction in the bandgap. Such changes increase the DOS near the Fermi level, thereby enhancing electronic conductivity and facilitating faster electron transport critical features for LIB performance [24]. Creating vacancies in graphene introduces localized DOS near defect sites, which stabilizes the structure, lowers diffusion barriers, and enhances lithium interaction. Doping with non-metals (e.g., N, boron (B)) or metals (e.g., nickel (Ni), cobalt (Co)) further increases the DOS near the Fermi level, improving conductivity and charge transfer. Metal dopants introduce new energy levels, while non-metals strengthen Li–C bonding. Co-doping combines these effects, yielding porous, conductive structures with enhanced electrochemical performance [36,37]. Functionalization with oxygen (O)-containing groups and decoration with metal nanoparticles (NPs) such as silicon further improves lithiation dynamics, cycling stability, and capacity retention [8]. Additionally, composites of graphene with metal oxides or sulfides combine graphene’s conductivity with the high lithium storage capacity of the other component, optimizing overall performance [38]. DFT allows detailed investigation of these modifications by calculating charge distribution, band structure, and DOS, which are all crucial to understanding and enhancing electrode behavior [24]. For instance, Zheng et al. [39] examined the DOS and projected DOS (PDOS) and found that Li^+^ bonds with C atoms at graphene vacancies, disrupting the Dirac point by concentrating electrons at defect sites. Shamim et al. [40] employed the Generalized Gradient Approximation—Perdew–Burke–Ernzerhof (GGA-PBE) approach to calculate the DOS of nitrogen-doped graphene oxide (NDGO) before and after Li^+^ adsorption. Their results showed that Li^+^ absorption eliminates the small bandgap, increases the number of available electronic states (notably between −45 and −50 eV), and enhances conductivity beneficial for LIB operation. Beyond C-based materials, transition metal oxides like manganese dioxide (MnO_2_) are explored due to their high energy density and affordability [41]. Wu et al. [42] simulated 2D MnO_2_/graphene hybrid structures using SIESTA computational code, which models core–valence interactions but does not account for van der Waals (vdW) forces. The PDOS indicated electron donation from Li^+^ to the conduction band, with the Li 2s level above the MnO_2_/graphene conduction band maximum (Figure 3a). Strong C–O interactions gave the hybrid electrode a metallic character, naturally enhancing Li^+^ adsorption. Zhang et al. [43] investigated a λ-MnO_2_/graphene composite and found that graphene integration significantly enhanced electronic conductivity by reducing the bandgap from 1.079 eV to 0 eV. DOS and PDOS analyses (Figure 3b,c) showed increased hybridization between manganese (Mn) 3d and O 2p states, promoting covalent bonding that facilitates Li^+^ insertion. Electron density maps (Figure 3d) further confirmed enhanced Mn–O orbital interactions, contributing to improved Li^+^ diffusion and electronic properties.

### 2.3. Adsorption Kinetics

**Figure 4:**
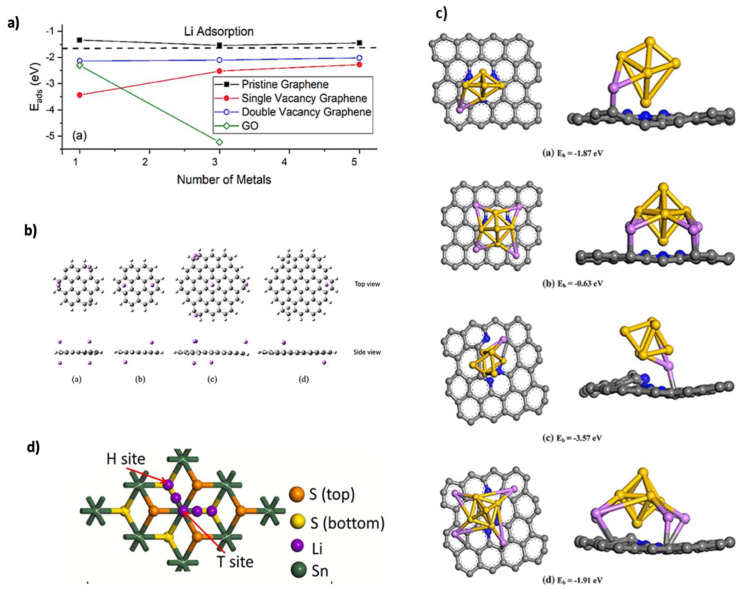
(**a**) E_ad_ of Li on different supports. Reproduced with permission [45]. Copyright 2019, MDPI. (**b**) Li-ion adsorption on C_24_H_12_ and C_54_H_18_ at q = 0 and −1: (**a**) 4 ions/C_24_H_12_ (−1); (**b**) 2 ions/C_24_H_12_ (0); (**c**) 5 ions/C_54_H_18_ (−1); and (**d**) 3 ions/C_54_H_18_ (0). Reproduced with permission [49]. Copyright 2020, Elsevier. (**c**) Optimized Li adsorption on N-DG: (**a**,**b**) pyridinic sites with single and four Li atoms; (**c**,**d**) pyrrolic sites with single and four Li atoms. Reproduced with permission [50]. Copyright 2018, Elsevier. (**d**) Adsorption site of Li on SnS_2_. Reproduced with permission [52]. Copyright 2021, Elsevier.

Adsorption kinetics significantly affect battery capacity and performance, playing a crucial role in the electrochemical processes of battery materials [44]. Stable adsorption enhances storage capacity by facilitating Li^+^ ion interaction with electrode surfaces and bulk diffusion, impacting the speed and efficiency of ion transport during charge–discharge cycles. These kinetics depend on intrinsic properties like surface morphology, electronic structure, and active sites. DFT simulations indicate that defects such as vacancies or dopants enhance adsorption energy by creating stable ion-binding sites, which improve ion diffusion and storage capacity. However, balancing adsorption strength and reversibility is challenging, especially with varying charge states and defect types. DFT calculations generally assume static conditions and cannot fully capture the dynamic ion behavior during cycling. Adsorption energy (E_ad_) is calculated as follows:

$$E_{ad}=E_{nLi+sub}-E_{\left(n-1\right)Li+sub}-E_{Li}$$

where n is the number of Li adsorbed on the substrate, E_nLi+sub_ is the total energy of the nLi adsorbed substrate, E_(n−1) Li+sub_ is the total energy of the substrate with (n − 1) adsorbed Li atoms, and E_Li_ is the energy per atom in large quantities of Li [22]. Dimakis et al. [45] found that Li adsorption on pristine graphene is unstable, but defects such as vacancies, substitutional dopants, and edge dislocations stabilize adsorption by creating additional electronic states near the Fermi level. This modification accelerates Li^+^ ion diffusivity and enhances electronic conductivity, as shown in Figure 4a. Similarly, Zhang et al. [46] observed that oxygen vacancies in Lithium titanate (Li_4_Ti_5_O_12_)/titanium dioxide (TiO_2_) (B phase) anodes improved lithium storage capacity and cyclic stability. Graphene quantum dots (GQDs), with high surface-to-volume ratios, serve as promising anode materials for lithium-ion adsorption, maximizing active sites [47,48]. Pattarapongdilok et al. [49] investigated Li^+^ adsorption and mobilization on graphene quantum dots (GQDs) using M06-2X/6-31G(d) theory, which was selected for its ability to capture dispersion interactions and binding energies. They studied three GQD sizes (C_24_H_12_, C_54_H_18_, and C_96_H_24_), and charge states (−1 for charging, 0, and 1 for discharging) were considered. Adsorption energy depended strongly on both size and charge. For C_24_H_12_, the −1 state showed the highest adsorption energy (−135 kcal/mol), accommodating up to five Li^+^ ions, compared to a maximum of three for neutral or positively charged states. Figure 4b illustrates the resulting adsorption patterns across GQD sizes and charges. Doped graphene composites have been extensively studied to enhance lithium adsorption. Hu and Zhou [50] used the Cambridge Serial Total Energy Package (CASTEP) to investigate Si clusters stabilized in N-doped defect graphene, highlighting that pyridinic nitrogen creates additional intercalation sites facilitating stronger Li adsorption with shorter bond lengths at Si–Si bridge sites, making it the most effective nitrogen doping type. Among these, pyridinic nitrogen where nitrogen replaces a C atom at the edge of a graphene ring creates additional intercalation sites that facilitate Li adsorption, as shown in Figure 4c. This configuration optimizes lithium binding by favoring anchoring at specific sites, such as Si–Si bridge positions, resulting in shorter Li–substrate bond lengths and stronger interactions. Consequently, pyridinic nitrogen is the most effective type for lithium adsorption. Bijoy and Lee [51] examined WS_2_/graphene heterostructures and reported minimal lattice expansion during Li intercalation, with E_ad_ ranging from −2.33 to −1.99 eV for Li_x_ (x=2–6), and no Li clustering. B-doping increased adsorption energy to −3.12 eV, enhancing anode stability, compared to −2.09 eV for N-doping. Wang et al. [52] studied sulfur (S)- and N-doped graphene/tin disulfide (SnS_2_) anodes using first principles Vienna Ab initio Simulation Package (VASP) calculations with GGA-PBE and a 20 Å vacuum to avoid layer interactions. Both dopants improved ion diffusion and adsorption, increasing storage capacity. Figure 4d shows the Li adsorption site on SnS_2_, where interlayer adsorption energy between graphene and SnS_2_ reached 3.83 eV, which is higher than individual layers. Li adsorption outside the heterostructure was 2.50 eV, surpassing pure graphene’s 1.93 eV, indicating enhanced stability through charge transfer and stronger interlayer interaction.

### 2.4. Diffusion Kinetics

**Figure 5:**
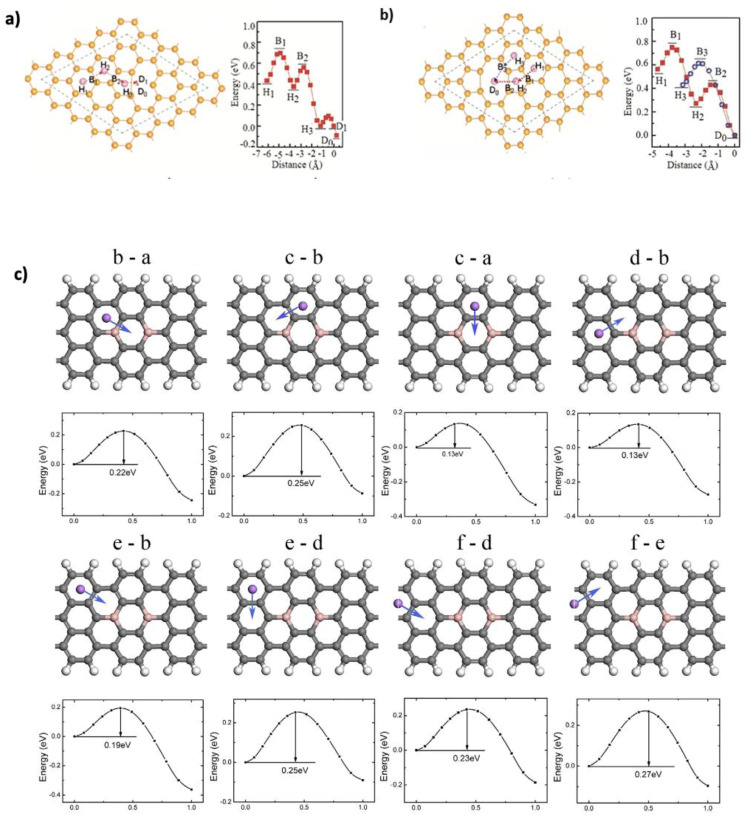
(**a**) Schematic representations and potential energy curves of Li diffusion on graphene double vacancy via H3–B3–D0 and H1–B1–H2–B2–D0. (**b**) Single vacancy via H1–B1–H2–B2–H3–D1–D0. Reproduced with permission [57]. Copyright 2012, American Chemical Society. (**c**) Diffusion barriers of Li on BGNRs. Reproduced with permission [22]. Copyright 2019, Elsevier.

The diffusion path of Li^+^ ions on graphene is crucial for reversible lithium storage and capacity. Efficient and low-energy diffusion pathways enable Li^+^ ions to intercalate and de-intercalate smoothly, maximizing the amount of lithium that can be stored and released repeatedly. If the diffusion path is hindered by high energy barriers or structural limitations, ion mobility decreases, resulting in lower reversible capacity and reduced battery efficiency [39]. Li^+^ diffusion on graphene is affected by factors such as defects (e.g., vacancies or Stone–Wales defects) and doping. Doping alters the local charge distribution and can reduce diffusion barriers. Among dopants, nitrogen is especially effective, as it creates electron-rich sites that facilitate lithium mobility [34,53,54]. At low lithium concentrations, the center of graphene hexagons is the most stable diffusion site, with minimal energy barriers when ions migrate between adjacent hexagons [55,56]. Fan et al. [57] investigated lithium diffusion on pristine and defective graphene using DFT. In pristine graphene, two symmetric paths were studied: Hex.–Bridge–Hex. (0.311 eV, 2.462 Å) and Hex.–Top–Hex. (0.337 eV, 2.844 Å). In bilayer graphene, the HT-HT path had the lowest diffusion barrier of 0.277 eV (1.421 Å). Defects, particularly double vacancies, further reduced the diffusion barriers to 0.17 eV, as shown in Figure 5a,b. These defects enhance Li^+^ mobility by allowing ions to pass through vacancy centers, increasing accessibility to the graphene surface [58]. Uthisar and Baron [59] examined the effect of graphene edges on Li^+^ diffusion using Amsterdam Density Functional (ADF-2012) package. Three paths were analyzed: through the center of the hexagon (H), the middle of the bond (M), and over a C atom (T). Narrow graphene nanoribbons showed lower barriers near edges (0.28 eV), compared to pristine graphene. Liu et al. [22] studied boron-doped graphene nanoribbons (BGNRs) using the DMol^3^ program.

The calculation of diffusion barrier values was based on the testing of all possible diffusion sites between two adjacent sites, as shown in Figure 5c. The diffusion barriers ranged from 0.13 to 0.27 eV, compared to 0.26–0.34 eV in undoped graphene nanoribbons (GNRs), confirming that B-doping facilitates Li mobility. Additionally, Kong et al. [60] found that Si doping decreases diffusion barriers in defective graphene from 0.2639 to 0.1333 eV. Wang et al. [52] explored S- and N-doped graphene/SnS_2_ heterostructures, finding enhanced Li^+^ mobility with a diffusion barrier of 0.21 eV—lower than that of pristine graphene (0.30 eV) and SnS_2_ (0.25 eV). Moreover, applying an external electric field can further reduce diffusion barriers. Tran et al. [61] demonstrated this effect in penta-graphene nanoribbons via DFT, showing improved Li-ion transport while maintaining high conductivity. These results suggest that combining defect engineering, doping, and external fields can significantly enhance the diffusion performance of graphene-based anodes. DFT provides valuable insights into Li^+^ diffusion in graphene, but its predictions are limited by system-specific results that often do not translate to real-world conditions. Dopants (e.g., B, N, and Si) and external fields can lower diffusion barriers, yet their effects depend on factors like concentration and defect interactions and remain difficult to validate or scale experimentally.

### 2.5. Challenges

DFT is a powerful tool for probing atomic-scale phenomena in battery materials but faces challenges when applied to complex systems like electrodes, electrolytes, and interfaces due to high computational demands. It also struggles to predict the composition and stability of lithium-rich phases, particularly in materials with large volume changes—key to determining theoretical capacity [62]. Time-Dependent DFT (TDDFT), an extension of DFT, addresses some limitations by enabling the study of electronic excitations and dynamic processes. Unlike conventional DFT, TDDFT projects Kohn–Sham equations into a reduced Hilbert subspace, facilitating accurate calculations of excitation energies and ionic forces with improved efficiency [63]. For instance, Ajeel et al. [64] used TDDFT to calculate the excitation energy (E_ex_), absorption wavelengths (μ_max_), electronic transition assignments, and optical band gap (E_og_) of graphene nanostructures doped with lithium impurities. Ardakani and Morad used TDDFT with turbo-eels to compute the optical properties of graphene with 2%, 3%, 5.5%, and 12.5% selenium (Se) impurities [65]. Conventional DFT often underestimates electronic correlation effects, causing inaccuracies in predicting battery performance. Generalized Gradient Approximation (GGA) and Local Density Approximation (LDA) provide basic approximations but suffer from self-interaction errors leading to electron delocalization. This is problematic for materials with localized d-electrons, where DFT may incorrectly predict metallic ground states, voltages, and redox behavior. These limitations highlight the need for more accurate methods or correction schemes [66,67]. DFT+U improves electron localization by incorporating on-site Coulomb interactions into the exchange-correlation functional. It maintains computational efficiency while enhancing descriptions of electrons in d, f, and localized p orbitals [68,69]. The Hubbard U correction yields more accurate predictions of charge transfer, redox reactions, and electronic structures critical for designing high-performance battery electrodes [70,71]. Many researchers used the VASP with Hubbard U to model the structural and electronic properties of battery electrodes [72]. Morgan et al. showed that standard GGA in the VASP is insufficient, whereas GGA+U restored gap states seen in photoelectron spectra [73]. Zhang et al. emphasized the necessity of GGA+U for accurate electronic properties of defective 3d transition metal–graphene/WSe_2_ heterostructures [74]. The choice of exchange-correlation (XC) functional significantly affects DFT accuracy. Devi et al. demonstrated that Strongly Constrained and Appropriately Normed (SCAN) generally yields lower mean absolute errors and more accurate results than other XC functionals, making it suitable for battery material simulations, though SCAN is computationally more expensive and sometimes faces convergence challenges [75]. Semi-empirical dispersion corrections, like Perdew–Burke–Ernzerhof (PBE) combined with Grimme’s D3 correction (PBE+D3), handle long-range interactions but show limitations in systems with mid-range vdW interactions. These include coordination errors, which are problematic for modeling adsorption on surfaces and interfaces of battery materials [76,77]. Grimme et al. [78] emphasized PBE+D3 underperformance, while SCAN offers more reliable modeling through accurate mid-range vdW parameterization. Sun et al. [79] developed SCAN as a meta-GGA functional that captures electron density and exchange-correlation energy more accurately, outperforming PBE+D3 in mid-range vdW interactions by satisfying all 17 known constraints of meta-GGA functionals. Meta-GGA functionals like SCAN balance accuracy and computational cost enhance the modeling of defective or impurity systems without the heavy demands of hybrid functionals or many-body perturbation theories [80,81]. This makes them practical for studying complex battery materials. Lithium adsorption studies require precise electronic interaction calculations; SCAN improves predictions but ideal accuracy remains challenging without high computational cost. Still, SCAN and similar meta-GGA functionals provide a viable compromise between cost and precision in the DFT modeling of battery systems [80]. For graphene-based materials widely explored for next-gen LIB atomic-scale mechanisms like lithium intercalation, diffusion kinetics, and defect formation add complexity, influencing capacity, rate capability, and cyclability. The interplay of structural distortions, charge transfer, and local bonding during lithiation/delithiation complicates deriving general design principles from DFT. Hybrid functionals like HSE06 are desirable for capturing charge localization and redox dynamics with higher fidelity but remain computationally prohibitive for large or defect-rich systems. More efficient methods like GGA+U or SCAN meta-GGA help improve predictions of formation energies and lithium adsorption in graphene systems, balancing accuracy and feasibility [24,82]. However, even these require careful validation against experiments, especially given the notable discrepancies observed between theoretical predictions and experimental results for various graphene-based anode materials, as summarized in Table 1.

## 3. Graphene-Based Anode Materials for Lithium-Ion Batteries (LIBs)

### 3.1. Overview of Lithium-Ion Batteries and the Role of Graphene

With increasing demand for high-performance energy storage systems, significant efforts have been directed toward improving the electrochemical performance of LIBs. Key strategies include optimizing electrode materials, reducing ion diffusion distances, enhancing conductivity and porosity, and improving electrolyte ionic conductivity. These approaches aim to mitigate polarization effects and electrokinetic instabilities, particularly under high charge–discharge rates, where uneven ion distribution can impact battery efficiency [93].

Graphite has long been the standard anode material in lithium-ion batteries (LIBs) due to its low cost and stable cycling performance, but its limited theoretical capacity (372 mAh g^−1^) and poor rate capability constrain its potential for advanced energy storage. Graphene emerges as a promising alternative, offering a higher theoretical capacity (~744 mAh g^−1^), along with excellent electrical conductivity and mechanical flexibility, which contribute to improved battery stability and lifespan [6,38,94]. In comparison, high-capacity materials such as silicon, transition metal oxides, and metal sulfides exhibit much greater specific capacities but suffer from drawbacks like severe volume expansion, low intrinsic conductivity, and poor cycling stability. Table 2 provides a summary of the key advantages and limitations of these materials. Beyond its standalone performance, graphene’s two-dimensional architecture and superior conductivity make it an ideal matrix in composite systems, where it helps buffer structural changes and enhance charge transport, effectively mitigating the weaknesses of these high-capacity materials and improving the overall electrochemical performance of LIBs [38,94,95].

### 3.2. Engineering Graphene for High-Performance Anode Materials in LIBs

Graphene’s exceptional properties make it a prime candidate for enhancing LIBs. This section reviews the synthesis methods for graphene, which are crucial for producing high-quality graphene. Functionalization techniques, such as introducing porosity, creating edge defects, and managing intrinsic defects, are explored to optimize graphene’s performance as an anode material. These modifications enhance the material’s electrical conductivity, lithium-ion diffusion, and overall capacity. By addressing these advancements, this section aims to highlight the potential of functionalized graphene for achieving high-performance LIBs.

#### 3.2.1. Graphene Synthesis

**Figure 6:**
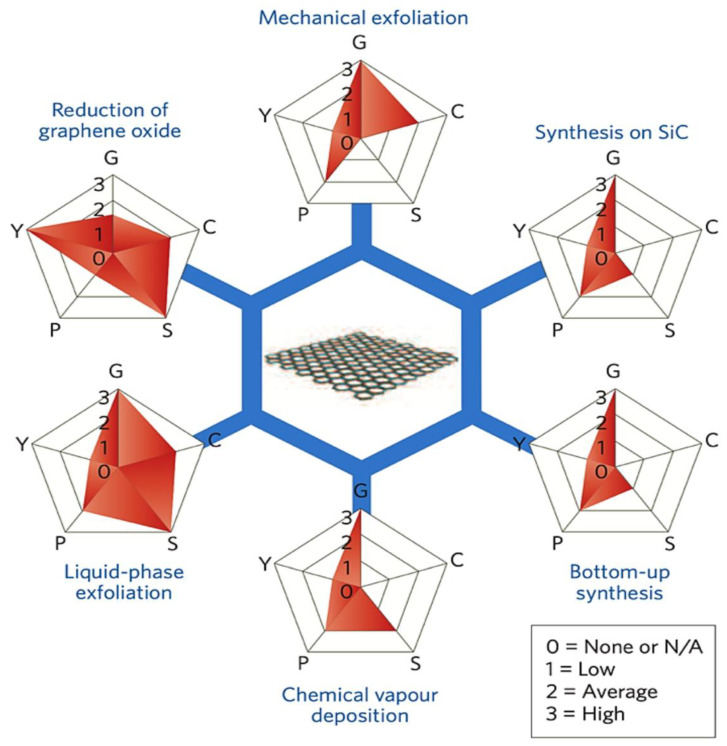
Schematic of the most common graphene production methods evaluated by graphene quality (G), cost (C), scalability (S), purity (P), and yield (Y). Reprinted with permission [10]. Copyright 2014, Springer Nature.

**Figure 7:**
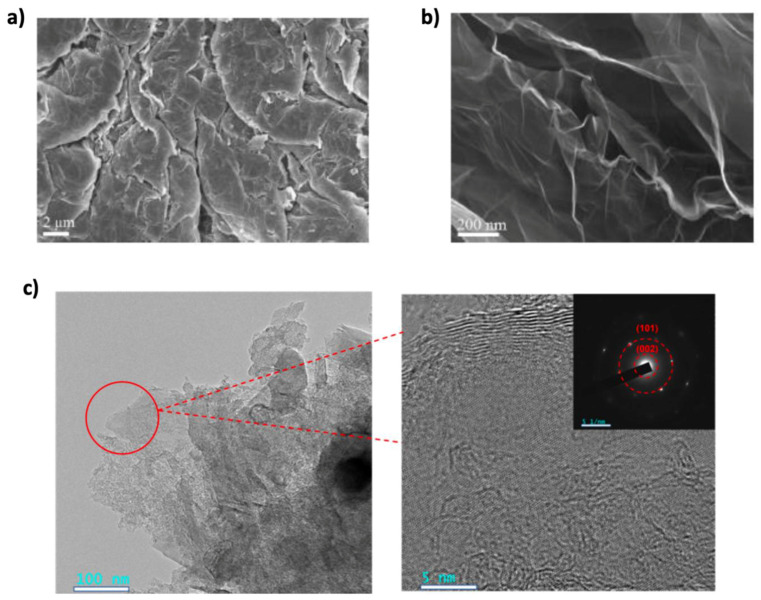
(**a**,**b**) SEM images of pristine graphite and exfoliated graphene sheet, respectively. Reprinted with permission [113]. Copyright 2017, Taylor & Francis Ltd. (**c**) TEM and Selected Area Electron Diffraction (SAED) images of carbonized BC/LEGr-I. Reproduced with permission [114]. Copyright 2023, MDPI.

Since 2004, researchers have focused on developing methods to synthesize graphene in large quantities for research purposes [96]. The synthesis method significantly impacts graphene’s quality, with top-down and bottom-up methods being the main categories [96,97]. Several methods have been proposed to synthesize graphene, including mechanical exfoliation (the “scotch tape” method), liquid-phase chemical/electrochemical exfoliation, unzipping carbon nanotubes, reduction of graphene oxide, chemical vapor deposition (CVD) on metal surfaces, and epitaxial growth on single-crystalline silicon carbide [98]. A comparative overview of these synthesis methods in terms of quality, cost, scalability, purity, and yield is illustrated in Figure 6. However, bottom-up methods like CVD and epitaxial growth face scalability challenges and high costs, making them less feasible for large-scale production [10]. Despite graphene’s remarkable properties and research advancements, large-scale, cost-effective integration into commercial batteries remains a significant challenge [99,100]. While high-quality graphene can be synthesized through various methods, scaling these processes to industrial levels is difficult. For instance, CVD requires specialized equipment with high energy consumption, making it costly and limiting scalability for battery applications. Additionally, non-carbon impurities introduced during the CVD process can negatively affect graphene’s electrical conductivity, impacting the electrochemical properties and cycling stability of graphene-based electrodes [101]. The mechanical exfoliation method is unsuitable for industrial graphene production due to its high cost and limited scalability. Instead, the reduction of GO is widely used, not only in energy storage but also in areas like biomedicine and nanotechnology. This method involves oxidizing graphite to produce GO and then reducing it to obtain graphene. Various reducing agents, including hydrazine, are commonly used. Hydrazine is one of the most efficient reducing agents, but it poses health and environmental risks if not disposed of properly [10,98]. To address these limitations, recent research has focused on optimizing CVD conditions such as temperature, pressure, and precursor materials to improve scalability while preserving graphene quality [102]. This has led to growing interest in green reduction methods using more environmentally friendly alternatives. Several plant extracts have been explored as green reductants including mushroom extracts [103], green tea [104], salvadora persica extract [105], coconut water [106], carrot juice [107], colocasia leaf and orange peel extracts [108], pomegranate juice [109], rose water [110], etc. Additionally, green reductases such as microorganisms, organic acids, and sugars have been studied [111]. In some cases, the reduction agent requires another substance or stabilizer to assist the reduction process or prevent platelet aggregation, and these materials are still under investigation for their effectiveness [112]. Liquid-phase exfoliation is one of the most widely used methods in combination with GO reduction. It involves dispersing graphite in a solvent to weaken van der Waals forces, followed by applying external forces such as ultrasound, electric fields, wet ball milling, shearing, or microfluidics to exfoliate it into graphene sheets. While this method offers better scalability, it typically produces lower-quality graphene, which can reduce battery performance [10,98]. To address this, several enhancements have been proposed. For instance, Shang et al. [113] combined supercritical carbon dioxide (CO_2_) with microjet exfoliation to produce high-quality graphene nanosheets. Scanning electron microscopy (SEM) images (Figure 7a,b) confirmed effective exfoliation, with 88% of the sheets having fewer than three layers and a relatively high conductivity of 2.1 x 10 S m^−1^. Similarly, Kewei Shu et al. [114] used bacterial cellulose as a green dispersant in liquid-phase exfoliation, producing graphene nanosheets with enhanced electrochemical activity. Transmission electron microscopy (TEM) images (Figure 7c) confirmed successful exfoliation and revealed folded structures that contribute to improved performance. Despite these advancements, the large-scale integration of graphene into lithium-ion batteries continues to face challenges including durability, consistent electrochemical performance, and compatibility with existing manufacturing infrastructure. Addressing these issues requires not only improving the quality and yield of synthesized graphene but also ensuring reproducibility and standardization across production methods. Among recent approaches, electrochemical synthesis stands out for its scalability, reduced environmental impact, and ease of integration with current battery technologies. Continued optimization of such methods is essential for translating lab-scale success into commercially viable energy storage solutions [102].

#### 3.2.2. Functionalization of Graphene

**Figure 8:**
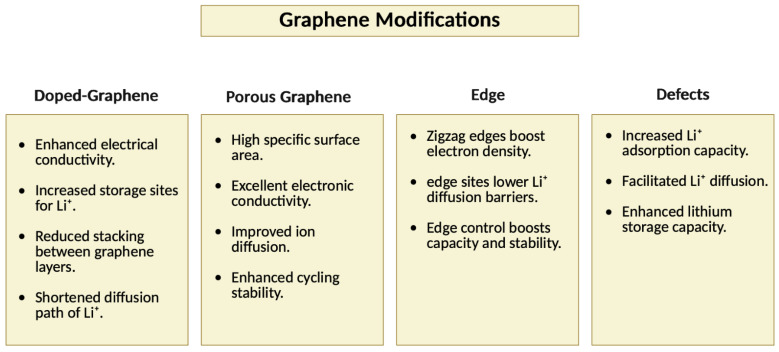
Summary of various graphene modifications including doping, porosity, edge engineering, and defects and their impact on lithium storage performance (this figure was created by the authors).

Graphene-based electrodes, though promising, are less commonly used due to the complex influence of their microstructure on ion storage and diffusion. To overcome these limitations, various chemical and physical modifications have been explored. Doping graphene with heteroatoms such as N, S, and B introduces functional groups that enhance ion adsorption, conductivity, and structural stability. For instance, nitrogen doping improves electrochemical performance by increasing active sites and stabilizing the material during cycling. In addition to doping, graphene is often combined with metal oxides and transition metal sulfides to form composite materials. While these materials typically suffer from issues like low conductivity and volume expansion, the incorporation of graphene helps buffer these effects, improving structural integrity and stability during lithiation cycles [115,116]. Section 3.3 further discusses the role of doping and composites in detail. This section also covers the role of porous graphene, edges, and defects in boosting performance. Porous structures and engineered edges increase surface area and ion accessibility, while defects create localized active sites, improving ion adsorption and overall battery performance. A summarized overview of these modification strategies and their influence on lithium storage is illustrated in Figure 8. These modifications collectively pave the way for more efficient and durable graphene-based anode materials in lithium-ion batteries.

##### Porous Graphene

**Figure 9:**
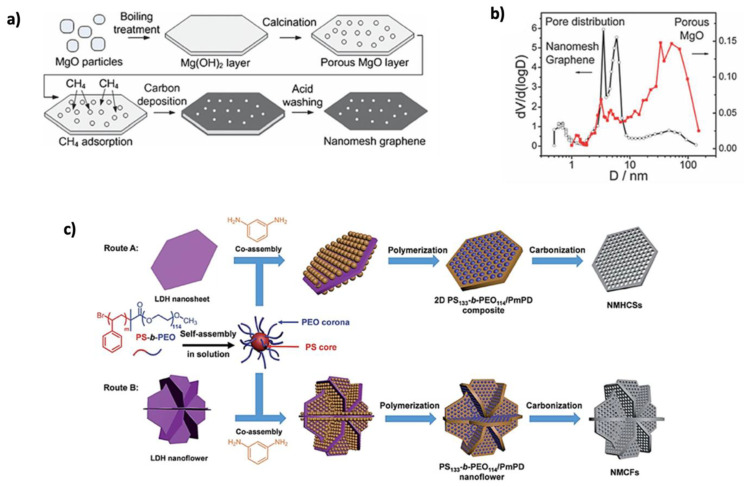
(**a**) Formation of polygonal nanomesh graphene. (**b**) Pore size distribution of porous MgO after boiling treatment and purified nanomesh graphene. Reproduced with permission [120]. Copyright 2023, Royal Society of Chemistry. (**c**) Schematic of dual-template self-assembly of NMHCSs and 3D N-doped carbon nanoflowers (NMCFs). Reproduced with permission [125]. Copyright 2023, Royal Society of Chemistry.

In addition to graphene’s remarkable advantages, porous graphene (PG) materials have gained significant attention due to their high specific surface area (SSA) and excellent electronic conductivity, greatly enhancing energy storage applications [117]. PG electrode materials improve ion transport, prevent volume changes, reduce initial irreversible capacity, enhance initial Coulombic efficiency (CE), and increase cycling stability [118]. The creation of pores in the graphene sheet, caused by the absence of C atoms, leads to porous graphene formation [119]. PG synthesis methods are categorized into template and template-free methods. Among the template methods, hard templates, such as porous silica and metal oxides, are the most widely used. These templates maintain their shape with minimal deformation during synthesis [117]. For high-quality porous graphene, magnesium oxide (MgO) is a suitable template. For example, Guoqing Ning’s group [120] prepared nanomesh graphene by template growth on porous MgO layers, as shown in Figure 9a. The MgO layers were created through boiling and calcination, forming Mg (OH)_2_ meshes with a thickness of 200–400 nm. After calcination at 500 °C, the graphene was synthesized on a gram scale with a C yield of 3–5 wt%. The resulting graphene had a surface area of 1654 m^2^ g^−1^ and a pore size of less than 10 nm, as shown in Figure 9b. Other inexpensive templates, such as Mg (OH)_2_ [121], Fe_3_O_4_ [122], and zinc chloride (ZnCl_2_) [123], have also been developed for producing PG. The hard-template method offers advantages like high surface area, porosity, and easy heteroatom doping, but it has drawbacks such as low graphene yield and complex post-treatment, often requiring hazardous chemicals [5]. In contrast, the soft-template method is simpler, using molecular precursors like amphiphilic macromolecular aggregates [117]. It offers better control over the thickness and pore structure of 2D materials and can be removed with non-toxic solvents or non-corrosive chemicals, making it a more convenient option [124]. Tian’s group [125] reported a dual-template strategy for the synthesis of N-doped mesoporous hexagonal carbon nanosheets with holey pores (NMHCSs). Polystyrene-b-poly (ethylene oxide) block copolymer was used as a soft template to construct the pores, layered double hydroxide (LDH) nanosheets or nanoflowers were used as a template to guide the sacrificial morphology, and m-phenyl-diamine (mPD) was used as a sacrificial morphology directing template; Figure 9c shows the synthesis steps. After testing, NMHCSs exhibited high electrical activity, with an average pore size of 14 nm and a SSA of 256 m^2^ g^−1^. Furthermore, Fang et al. [126] developed a new approach combining soft- and hard-template methods, which was developed to prepare a 2D porous C material. Using phenolic resol Pluronic composites as subunits and anodic aluminum oxide (AAO) membranes as a substrate, mesoporous graphene nanosheets (GNSs) were created with 9 nm-sized mesopores. Due to the thin nature of GNSs, mesopores form only in a single layer, resulting in a 2D structure. The GNSs demonstrated good anodic performance for Li^+^ storage, with a capacity of 1040 mAh g^−1^ at 100 mAh g^−1^ and 833 mAh g^−1^ after 75 cycles. While the soft-template method provides more flexibility in controlling morphology, it is less stable than the hard-template method [124]. Template-based preparation methods are generally more complicated and costly compared to template-free methods. In template-free methods, several techniques, such as thermal etching, wet chemical etching, and catalytic etching, are used to introduce defects into the graphene basal planes. However, this method lacks the ability to precisely control the formation and porosity of the resulting materials and often leads to severe corrosion and environmental pollution [117,124]. To overcome these trade-offs, hybrid synthesis strategies combining the structural precision of template methods with the simplicity of template-free approaches show promise. Using self-assembled biomolecules or supramolecular frameworks as removable templates could provide pore control and environmental compatibility. Additionally, integrating green chemistry principles, such as solvent-free synthesis, low-temperature activation, and biomass-derived C precursors, can reduce environmental impact. Additionally, predictive computational modeling can assist in designing hierarchical pore structures tailored to specific battery chemistries.

##### Edge

**Figure 10:**
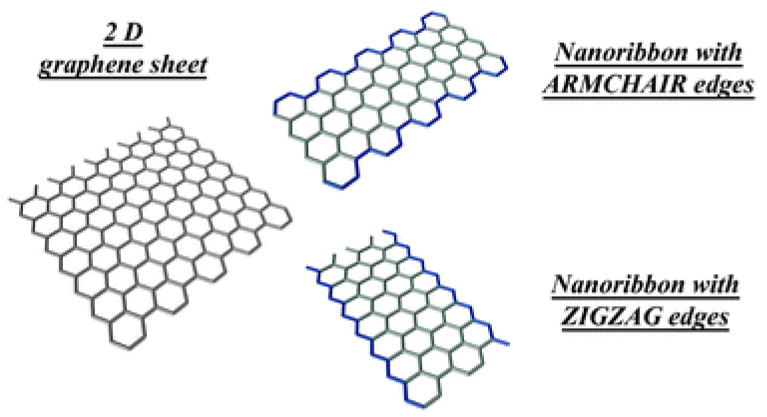
Graphene structures with zigzag and armchair edges. Reprinted with permission [127]. Copyright 2010, Royal Society of Chemistry.

Research into graphene, especially graphene edges (graphene ribbons), has grown rapidly in recent years due to its unique electronic, chemical, and magnetic properties [127]. The edges of graphene play a key role in its electronic and chemical interactions [128]. These edges can take forms like zigzag, armchair, or a combination of both (Figure 10) [129]. The edge form significantly affects graphene’s electronic properties. Zigzag edges theoretically host unbound electron states, increasing the local density of states near the Fermi energy [128], while armchair edges feature C atoms forming triple covalent bonds, stabilizing chemical reactions [130]. Lithium storage is strongly influenced by edge sites in graphene, which act as defect sites with stronger interaction and adsorption with lithium-ions than the ideal graphene lattice [129]. Uthaisar and co-workers found that these edge sites play a crucial role in lithium-ion absorption and diffusion, as demonstrated by their DFT calculations [59]. When graphene is reduced to one dimension, both armchair and zigzag edges appear. It was observed that the energy barrier at these edges is lower by up to 0.15 eV compared to that of the graphene lattice, which enhances ion diffusion and improves battery performance. Among the two edge configurations of GNRs, the zigzag configuration has a stronger effect on performance than the armchair configuration. Narrower GNRs show faster discharge power performance due to reduced energy barriers and shorter diffusion lengths, particularly in the zigzag shape. Bhardwaj and co-workers reported the use of GNRs as an anode material for LIBs [131]. To fabricate GNRs, they used multi-walled C nanotubes (MWCNTs) with diameters ranging from 50 to 80 nm, combined with a solution-based oxidative technique. This study found that oxidized GNRs (ox-GNRs) exhibited superior initial capacity (1400 mAh g^−1^) compared to reduced GNRs, likely due to the presence of oxygen-containing guest groups. However, the CE of ox-GNRs was only 53% during the initial cycles. Both ox-GNRs and reduced GNRs were tested for their cyclability, with the average capacity loss per cycle being 3% for ox-GNRs and 2.6% for reduced GNRs. Xiao and co-workers studied the electrochemical performance of GNRs at various unzipping levels [132]. They evaluated GNRs at 5 min, 5 h, 10 h, and 20 h of unzipping. After 5 min, the C nanotubes retained their tubular structure with some surface cracks. The GNR 5 h sample showed a significant surface area increase (321.6 m^2^ g^−1^) and a discharge capacity of over 500 mAh g^−1^, which is higher than the GNR 10 h and GNR 20 h samples, indicating that performance decreases with increased unzipping time. Additionally, GNRs with oxygen-containing functional groups (GONRs) showed poor conductivity and larger irreversible capacity, leading to capacity fading in subsequent cycles. Rather than broadly optimizing surface area through prolonged unzipping, which may degrade structural integrity, future efforts should focus on precision edge tailoring—achieving an optimal balance between edge exposure and stability. Techniques like anisotropic etching, edge-selective functionalization, or bottom-up synthesis from molecular precursors could allow control over edge length, type, and chemical termination.

##### Defects

Graphene’s structural and topological defects can enhance its performance by acting as electroactive or stabilization sites for active species [97]. These defects, including point defects like zero-dimensional (0D) vacancies and interstitial atoms, can be single defects, Stone–Wales defects, Wells defects, or multiple defects. Defects in graphene arise from three main mechanisms: crystal growth, irradiation with active molecules, and chemical treatment. Defects in graphene play a crucial role in enhancing lithium-ion storage by providing energetically favorable adsorption sites that increase charge transfer and improve lithium binding. These defects lower diffusion barriers along certain pathways, facilitating Li^+^ ion transport within the electrode. However, excessive Li^+^ accumulation near defect sites may cause steric hindrance, which can limit ion mobility. Controlling defect density is important, as too many defects can increase irreversible lithium loss and voltage hysteresis, adversely affecting energy density and cycle life [129]. DFT calculations by Datta and co-workers confirmed that lithium-ion adsorption is enhanced in graphene with vacancies and Stone–Wales defects [84]. Their study found that defects increase the likelihood of lithium adsorption, particularly near defective regions, improving the adsorption rate and specific capacity as defect density increases. Cheng et al. [133] investigated the adsorption and diffusion of Li-ions in graphene with vacancy defects of varying sizes, labeled as GVn, where n represents the number of missing C atoms (2, 4, 6, 10, and 13). They found that lithium adsorption increases as defect size grows, and adsorption energies are higher near vacancy defects. However, the Li diffusion barrier increases on GVn surfaces (n = 6, 10, 13), as Li^+^ adsorption in larger defects is much stronger, making ion release difficult. The diffusion barrier decreases when vacancy defects are filled with lithium. The calculated storage capacities for GV10 and GV1 were 614 mAh g^−1^ and 637 mAh g^−1^, respectively, demonstrating a significant increase in lithium storage capacity with larger vacancy defects. Bulusheva et al. [83] introduced 2–5 nm holes in graphene layers to enhance LIB rate capability. These were formed by treating GO with concentrated sulfuric acid, followed by annealing at 1000 °C in argon. The acid-etched defects and annealing removed oxygen, creating pores that reduced capacitance loss and facilitated ion intercalation. The HG1000 sample delivered 210 mAh g^−1^ at 50 mA g^−1^, attributed to reduced electrolyte decomposition, improved Li^+^ adsorption at hole edges, and easier ion diffusion. Additionally, Dong and co-workers [134] produced graphene containing multiple vacancy defects through flash joule heating (FJH) technology. This method effectively reduces oxygen content and forms 3D carbon structures with intrinsic defects. The resulting defects enhanced cycling capacity and reduced dendrite formation, improving overall performance. The material reached a high capacity of 2450 mAh g^−1^ after 1000 cycles.

The next step is to explore ways to selectively introduce defects that maximize ion storage while reducing diffusion limitations. For example, controlled FJH could optimize defect size by tuning the energy input, enhancing ion adsorption without significantly hindering diffusion. Smaller, isolated vacancies could improve Li^+^ adsorption while minimizing diffusion barrier increases, balancing capacity, and conductivity for better control over material performance.

### 3.3. Graphene-Based Anodes: Materials and Composites

Graphene has emerged as a leading material in the development of LIBs due to its remarkable properties, including its large theoretical capacity, excellent mechanical strength, high surface area, and superior conductivity. Direct use of graphene as an anode material in its pristine form presents certain challenges, such as ionic-steric effects and potential instability under extreme cycling conditions. To address these limitations, extensive research has focused on developing graphene-based composites. These composites combine graphene with other materials to enhance its performance, providing the necessary structural integrity, ionic conductivity, and better cycle stability [6,8]. This section will explore the most important graphene-based composites and their positive outcomes as high-power anodes for LIBs, offering improved efficiency, charge–discharge rates, and overall battery life.

#### 3.3.1. Pristine Graphene

**Figure 11:**
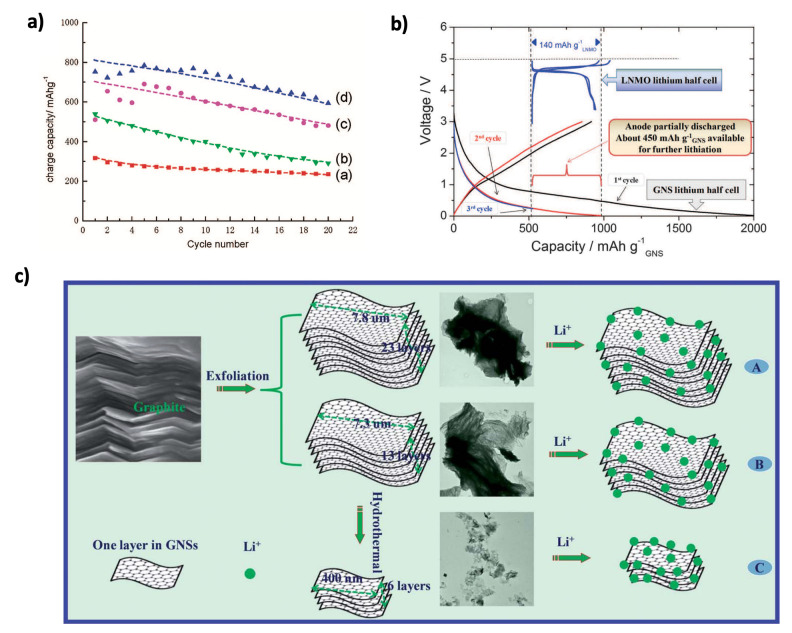
(**a**) Charge–discharge curves at 0.05 A g^−1^ for (a) graphite, (b) GNSs, (c) GNSs + CNTs, and (d) C_60_. Reprinted with permission from [6], 2022, MDPI. (**b**) Charge–discharge curves of GNSs at 74 mA g^−1^. Reproduced with permission [137]. Copyright 2023, Royal Society of Chemistry. (**c**) Morphological evolution and lithium storage of GNS-I, GNS-II, and GNS-III. Reproduced with permission [138]. Copyright 2014, Royal Society of Chemistry.

The theoretical specific capacity of graphene is 744 mAh g^−1^, which is twice that of graphite (372 mAh g^−1^). Due to its unique electronic structure, high charge carrier mobility (20 m^2^ V^−1^ s^−1^), high theoretical surface area (2630 m^2^ g^−1^), and broad electrochemical window, graphene has garnered significant interest in energy production and storage applications, particularly in LIBs [6,135]. One of the first reports on graphene as an anode material for lithium batteries was published in 2008 by Yoo et al. [136]. They compared the performance of graphite, GNSs, GNSs combined with carbon nanotubes (CNTs), and GNSs combined with fullerenes (C_60_). The reversible capacity values at a current density of 0.05 A g^−1^, as shown in Figure 11a, for graphite, GNSs, GNSs+CNTs, and GNSs+C_60_ were 320, 540, 730, and 784 mAh g^−1^, respectively. After 20 cycles, the capacity retention percentage for graphite was 78%, while for GNSs, GNSs+CNTs, and GNSs+C_60_, it was 54%, 66%, and 77%, respectively. These results indicate that while graphite experiences less capacity fade, GNSs and their composites exhibit larger capacities, suggesting higher lithium-ion insertion/extraction compared to graphite. In a related study, Vargas et al. [137] activated the GNS electrode before cycling, followed by cycling the lithium nickel manganese oxide (LNMO)-GNS whole cell (Figure 11b). This process ensures that the GNS electrode can effectively intercalate lithium-ions and prevents polarization caused by cell imbalance. The GNS electrode initially recorded a capacity of 2000 mAh g^−1^, which decreased to approximately 600 mAh g^−1^ after 50 cycles. The significant capacity loss was attributed to the decomposition of the electrolyte, leading to the formation of a thick SEI layer. Li et al. [138] synthesized three types of GNSs (GNS-I, GNS-II, and GNS-III) with varying morphologies (Figure 11c), prepared using a modified Hummers method. A reduced number of GNS layers, increased defects, increased edge sites, and smaller sizes were found to improve the performance and capacity of LIBs. The GNS-III electrode, produced by hydrothermal treatment, showed an initial discharge capacity of 2561 mAh g^−1^, which significantly dropped to 691 mAh g^−1^ after 100 cycles. In contrast, the GNS-I and GNS-II electrodes had initial discharge capacities of 925 and 418 mAh g^−1^, respectively, with values decreasing to 269 and 400 mAh g^−1^ after 100 cycles. Pristine graphene offers great potential with its high theoretical capacity, but its practical use in lithium-ion batteries is limited by long-term cycling stability issues, particularly SEI layer instability. To address graphene’s capacity loss over cycles, further advancements are needed to enhance its durability. Integrating graphene into composites or applying surface modifications could stabilize the SEI layer and improve lithium-ion intercalation, unlocking its full potential as a high-performance, durable anode material.

For a comprehensive overview, Table 3 summarizes the electrochemical performance parameters of pristine graphene alongside doped graphene and composite anodes, providing a consolidated reference for comparing their respective advantages and challenges.

#### 3.3.2. Doped Graphene

**Figure 12:**
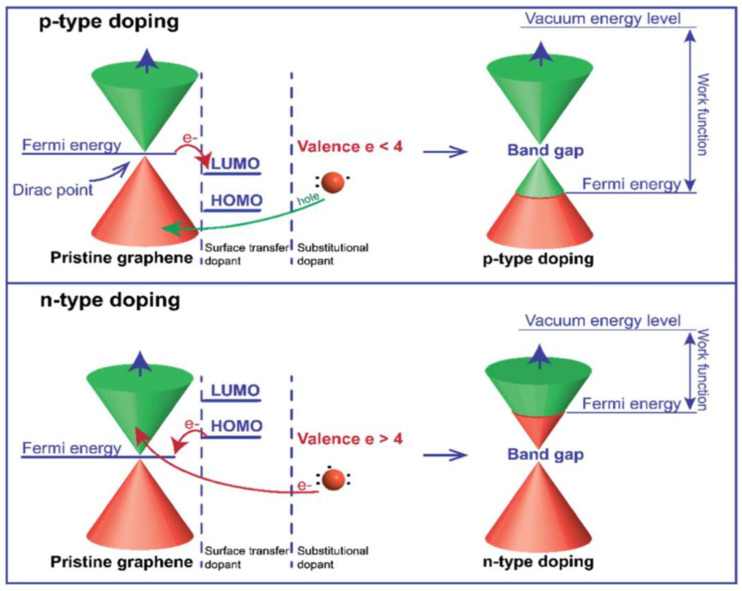
Illustration of the energy diagram of p-type and n-type doped graphene. Adapted with permission [36]. Copyright 2021, Royal Society of Chemistry.

Despite graphene’s outstanding properties, its limited active sites, specific capacitance, and electrical conductivity can affect performance as an anode material for LIBs [181]. Chemical doping (e.g., N, B, or S) can address these issues by enhancing electron and ion transfer [182]. Pristine graphene, being a gapless semiconductor, has limited control over its electronic structure. Chemical doping shifts the Fermi level depending on the dopant type, enabling a tunable band gap (Figure 12). For instance, B-doping introduces electron deficiency (p-type), while N-doping introduces extra electrons (n-type), both modifying charge distribution and improving transport properties [36,37].

**Figure 13:**
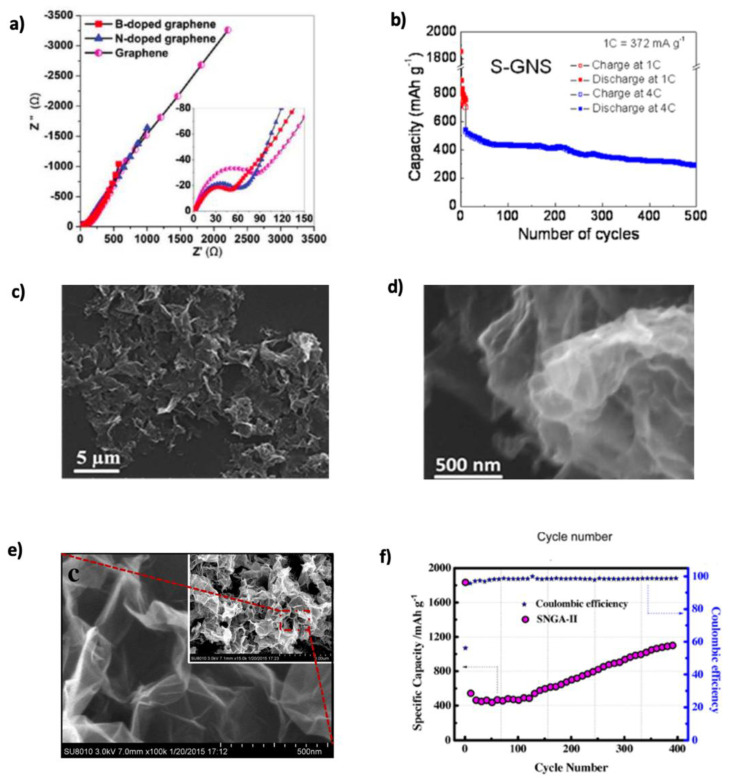
(**a**) EIS of pristine, N-doped, and B-doped graphene. Reproduced with permission [85]. Copyright 2011, American Chemical Society. (**b**) Cycling performance of S-GNSs for 500 cycles. Reprinted with permission [143]. Copyright 2014, Elsevier. (**c**,**d**) SEM images of NBGs-1000. Reproduced with permission [145]. Copyright 2015, Royal Society of Chemistry. (**e**) SEM images of SNGA-II. (**f**) Cycling performance of SNGA-II at a current density of 800 mA g^−1^. Reprinted with permission [147]. Copyright 2016, Elsevier.

Wu’s group [183] investigated lithium-ion adsorption and diffusion on doped graphene through DFT calculations. They found that B-doped graphene enhanced lithium absorption due to boron’s electron-deficient nature, whereas N-doped graphene exhibited lower adsorption but faster lithium diffusion. However, B-doping also increased the energy barrier for diffusion. To overcome this, Hardikar et al. [184] introduced structural defects such as mono-vacancies and Stone–Wales defects, reducing the diffusion barrier from ~0.73 eV to ~0.13 eV. These modifications enhanced lithium mobility and electrochemical performance. Zhou et al. [185] confirmed that B-doped graphene outperforms pristine and N-doped graphene in LIBs, achieving a theoretical capacity of 425.2 mAh g^−1^ due to stronger Li–C interactions. Shuai et al. [85] compared pristine, N-, and B-doped graphene, showing reversible capacities of 1043 and 1549 mAh g^−1^ for N- and B-doped samples, respectively, with improved retention over 30 cycles. Figure 13a shows reduced charge transfer resistance from N/B-doping due to defect-induced conductivity enhancement. Improved CE suggests partial suppression of electrolyte decomposition and stable SEI film formation. S, with a larger atomic radius (~1.78 Å), enhances Li^+^ adaptability and storage kinetics. Although sulfur tends to form linear nanodomains in graphene, it enhances Li^+^ storage kinetics [5]. Yun et al. [143] synthesized S-GNSs, which exhibited a discharge–charge capacity of 1700/870 mAh g^−1^ at 374 mA g^−1^. Their performance exceeded that of undoped graphene (400 mAh g^−1^), indicating superior conductivity and Li-ion transport. Figure 13b illustrates the long-term stability of S-GNSs. Conductivity measurements (1743 vs. 32 S m^−1^ for GNSs) further confirmed the enhanced charge carrier mobility via σ=neμ, where σ is the conductivity, n is the charge carrier number, e is the electron charge, and μ is the charge carrier mobility [186].

To further improve performance, co-doping strategies have been explored. Nitrogen, due to its structural versatility (e.g., pyridinic, graphitic), often co-dopes with other atoms to facilitate Li^+^ intercalation [5,183,187]. Huang’s group, [145] fabricated NBGs-1000, as seen in SEM images (Figure 13c,d). The crumpled architecture accommodates volume changes and introduces beneficial defects. Co-doping leverages N-induced surface area and conductivity, while B improves adsorption and expands interlayer distance. NBGs-1000 achieved a reversible capacity of 909 mAh g^−1^ and retained 96.5% after 125 cycles. Cai et al. [146] synthesized N and S co-doped graphene with 1016 mAh g^−1^ initial capacity, retaining 788.2 mAh g^−1^ after 50 cycles. Even at 20 A g^−1^, NSG delivered 250.1 mAh g^−1^, highlighting high-rate capability. Shan et al. [147] developed SNGA-II using a hydrothermal method; SEM images (Figure 13e) confirmed that porosity was preserved. SNGA-II retained 1109.8 mAh g^−1^ after 400 cycles with ~100% CE (Figure 13f). Feng et al. [188] supported these findings with DFT calculations. Charge density analysis showed that N atoms gained electrons while neighboring S/C lost electrons due to electronegativity differences. PDOS revealed enhanced states near the Fermi level, improving Li adsorption. Sungur et al. [148] synthesized Si-N co-doped graphene via a solvothermal route, achieving 540 mAh g^−1^ at 0.5 A g^−1^, increasing to 578.75 mAh g^−1^ after 500 cycles. The material showed enhanced rate capability due to increased interlayer spacing and larger graphene sheets. Its capacity also improved with cycling, which was attributed to surface charge redistribution and SEI interactions. DFT calculations revealed how SiC_4_ and SiN_1_C_3_ active centers on the graphene surface modulate the charge at the interface.

In summary, doping graphene with heteroatoms enhances lithium storage by improving conductivity, diffusion, and adsorption. To fully leverage these benefits while avoiding structural instability, future efforts should focus on precise control of dopant type, configuration, and concentration. Co-doping especially with nitrogen and boron offers notable improvements in conductivity and Li-ion intercalation. Although sulfur doping boosts lithium kinetics, it requires careful control to prevent issues like nanodomain formation. Optimizing dopant combinations and concentrations could further improve the performance and stability of graphene-based anodes.

#### 3.3.3. Graphene-Based Composite Materials

##### Si–Graphene Composites

**Figure 14:**
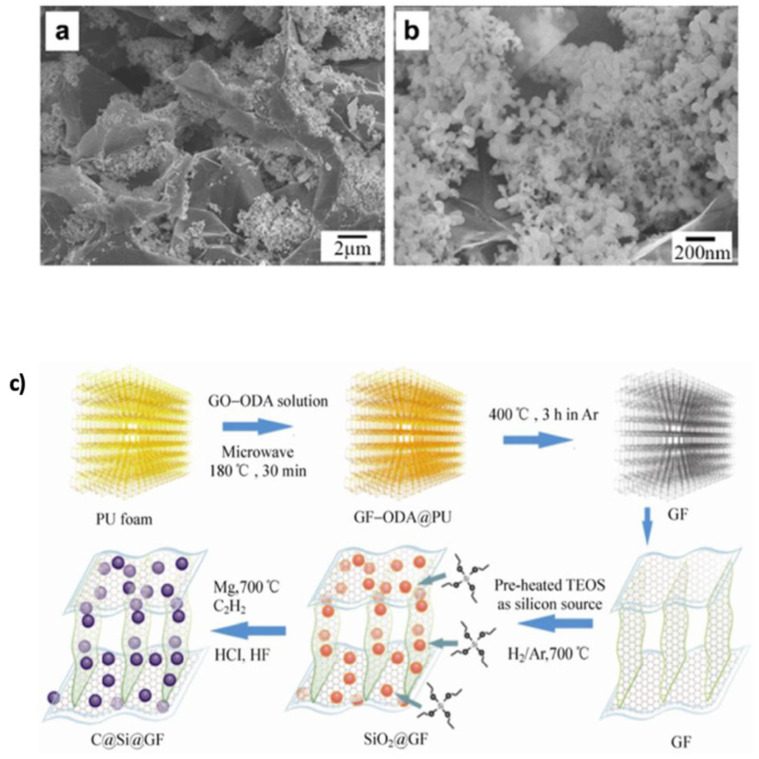
(**a**,**b**) SEM images of SGE. Reprinted with permission [88]. Copyright 2011, Elsevier. (**c**) Schematic illustration of the synthesis process for porous 3D Mesoporous Si@graphene foam (C@Si@GF). Reprinted with permission [149]. Copyright 2013, Springer Nature.

Si is one of the most promising alternative anode materials for LIBs due to its high theoretical capacity of 4200 mAh g^−1^ gravimetric and 9786 mAh cm^−3^ volumetric, which is approximately ten times that of conventional graphite anodes. In addition to its abundance, low cost, and environmental friendliness, Li–Si alloys possess high melting points, making them suitable for high-temperature applications [8,189]. However, Si suffers from severe volume expansion and cracking during lithiation, which leads to unstable SEI formation and rapid capacity decay. Integrating graphene into Si-based anodes offers a strategy to mitigate these drawbacks by providing a highly conductive and flexible matrix that accommodates volume changes and enhances charge transport [189]. Chou and Hwang [190] theoretically studied Si–graphene (Si–Gr) composites and found that graphene facilitates charge transfer and generates an interfacial electric field that attracts lithium cations, thus promoting faster Li-ion mobility and improving performance. Importantly, this enhancement occurs without significantly altering Si’s lithiation behavior, preserving its capacity and voltage profile. This framework is supported by Xiang et al. [88] who prepared graphene/silicon nanocomposites using reduced GO (rGO) and thermally expanded graphite. Nanosized Si particles filled the pores of expanded graphite (Figure 14a,b) and strongly adsorbed onto graphene sheets, enhancing electrical connectivity and ion transport. The composite achieved a specific capacity of 2753 mAh g^−1^ with 83% retention after 30 cycles, attributed to uniform Si dispersion and mechanical buffering by graphene.

**Figure 15:**
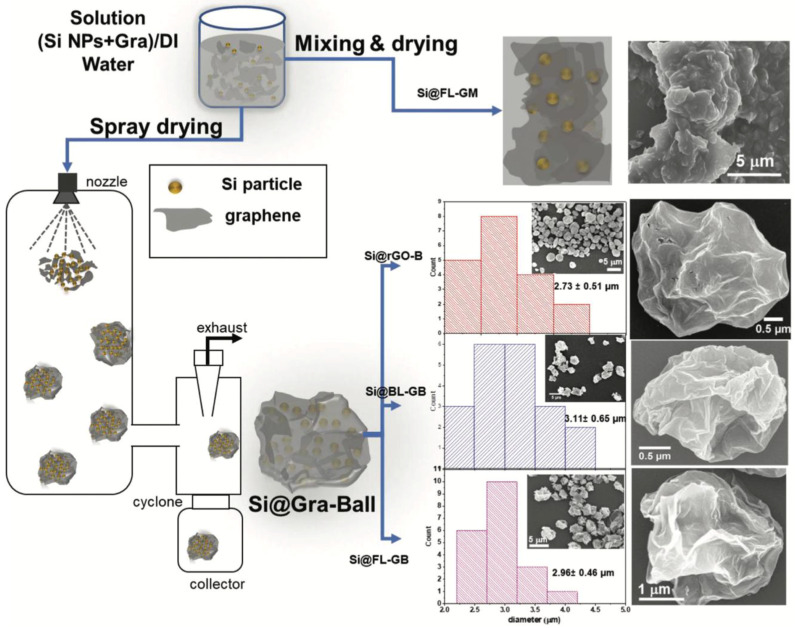
Schematic of the preparation of Si@Gra-balls via spray-drying. SEM images and particle size distributions of Si@rGO-B, Si@BL-GB, Si@FL-GB, and comparison with Si@FL-GM. Reprinted with permission [150]. Copyright 2020, Royal Society of Chemistry.

**Figure 16:**
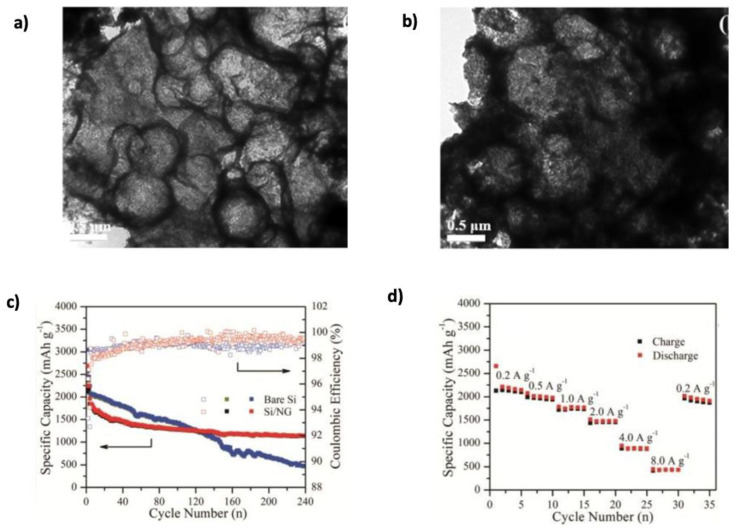
(**a**,**b**) TEM images of Sandwich-G/Si-2 before cycling and after 100 cycles. Adapted with permission [151]. Copyright 2020, Elsevier. (**c**) Comparative cycling performance of Si/NG and bare Si electrodes. (**d**) Rate capability at various current densities from 0.2 to 8.0 A g^−1^. Adapted with permission [153]. Copyright 2018, Royal Society of Chemistry.

Three-dimensional (3D) graphene–Si frameworks have shown further performance improvements. Chen et al. [149] developed a C@Si@GF composed of carbon Si–graphene nanostructures, which reduced the ion diffusion path and specific surface area (Figure 14c). This architecture achieved a capacity of 1200 mAh g^−1^ at 1 A g^−1^, retaining 89.1% after 200 cycles. In contrast, the pure Si electrode degraded rapidly to 25.8 mAh g^−1^ after only 20 cycles. Jamaluddin et al. [150] designed spherical microstructures by spray-drying Si NPs with graphene nanosheets (Si@Gra), forming a core–shell configuration that improved interfacial conductivity, protected Si from volume changes, and allowed high lithium intercalation. This material demonstrated an initial capacity of 2882.3 mAh g^−1^ with 70.9% retention after 100 cycles (Figure 15). Similarly, Liu et al. [151] created hollow spherical composites where Si NPs were sandwiched between graphene shells. This design limited agglomeration, enhanced SEI stability, and provided void space to accommodate expansion. After 500 cycles, the composite maintained a capacity of 1085.6 mAh g^−1^ at 100 mA g^−1^, with minimal structural degradation observed in TEM images (Figure 16a,b). Heteroatom doping of graphene further boosts composite performance. For instance, nitrogen doping (N-doping) enhances Li-ion adsorption and storage, as demonstrated by Nie et al. [152] who fabricated mesoporous Si spheres encased in N-doped graphene cages (Si@graphene cage) via CVD and magnesiothermic reduction. The material delivered an initial discharge–charge capacity of 4000/2217 mAh g^−1^ at 100 mA g^−1^, maintained 890 mAh g^−1^ at 5 A g^−1^, and remained stable over 200 cycles. Chen et al. [153] produced N-doped graphene-like carbon (Si/NG) composites with porous silicon wafers, achieving 1138 mAh g^−1^ at 1 A g^−1^ over 240 cycles. The hierarchical pores (0.26 cm^3^ g^−1^, 153 m^2^ g^−1^ surface area) allowed for better Li-ion transport and volume accommodation (Figure 16c,d). Enhanced wettability and flux from N-doping, combined with the graphene cage’s mechanical resilience, accounted for the stable performance. Complementing this, a DFT study by Kolosov and Glukhova [191] on a porous graphene–CNT–Si hybrid predicted a 37% capacity increase compared to undoped systems, due to improved charge distribution and Li-ion filling behavior.

Despite these advancements, long-term stability challenges persist. While graphene effectively buffers Si expansion, sustainable performance requires more sophisticated architectures like hollow or multi-layered graphene frameworks. Precision strategies, such as uniform N/S co-doping and controlled dispersion of NPs, can further enhance ion mobility and structural resilience. Incorporating elastic matrices into hybrid designs may offer additional mechanical flexibility and improve cycling durability.

##### Transition Metal Oxide–Graphene Composites

**Figure 17:**
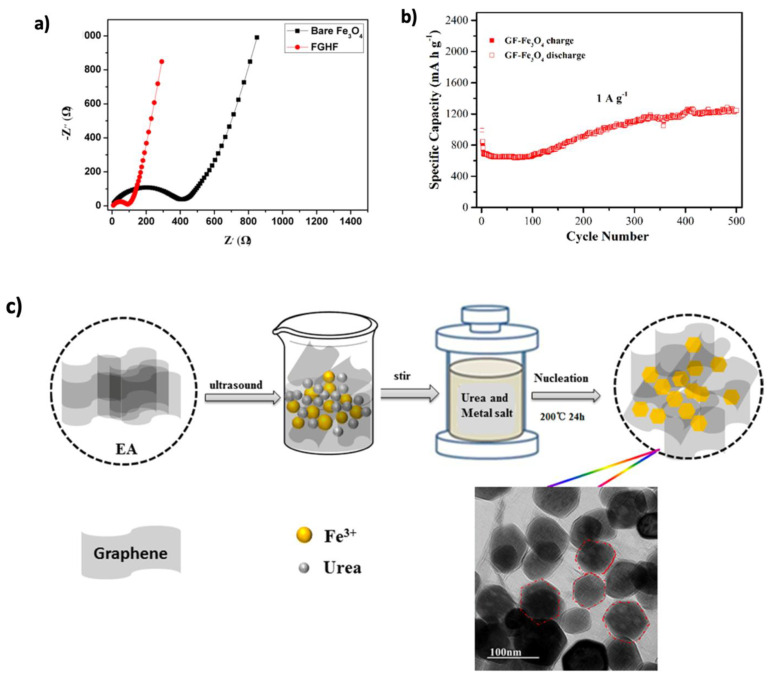
(**a**) EIS spectrum of the FGHF and bare Fe_3_O_4_ electrode. Reproduced with permission [154]. Copyright 2019, Elsevier, licensed under CC BY-NC-ND 4.0. (**b**) Cycling stability of GF-Fe_3_O_4_. Adapted with permission [157]. Copyright 2017, Elsevier. (**c**) Schematic of the fabrication process for the mesoporous Fe_2_O_3_/N-doped rGO composite. Reproduced with permission [158]. Copyright 2020, Elsevier.

Transition metal oxide (TMO) materials have drawn significant attention for improving the electrochemical performance of LIBs. Graphene, known for its chemical stability, electrical conductivity, and mechanical flexibility, has been combined with metal oxides to enhance their properties, including performance, stability, and volume change effects [38,94]. Iron oxides, particularly Fe_3_O_4_, are promising anode materials because of their high theoretical capacity, natural abundance, non-toxicity, environmental compatibility, and corrosion resistance [38,192,193]. DFT simulations by Glibo et al. [194] revealed a conversion-type lithiation mechanism in Fe_3_O_4_, where it transforms into metallic iron (Fe) and lithium oxide (Li_2_O) during discharge and reverts upon charging. While this contributes to its high capacity, issues such as volume expansion, poor cycling stability, and low conductivity persist. To overcome these, strategies like nanostructuring, C-based composites, and protective coatings have been proposed. Recent experimental studies have demonstrated the effectiveness of Fe_3_O_4_–graphene composites. Fang et al. [154] synthesized a sandwich-like Fe_3_O_4_/graphene hybrid film (FGHF) via electrostatic self-assembly and vacuum filtration. The Fe_3_O_4_ NPs were uniformly distributed between face-to-face stacked graphene layers, reducing charge transfer resistance and enhancing ion transport, as confirmed by electrochemical impedance spectroscopy (EIS) measurements (Figure 17a). The electrode delivered a high capacity of 947 mAh g^−1^ at 1 A g^−1^ and maintained 776 mAh g^−1^ after 300 cycles. Similarly, Li et al. [155] produced Fe_3_O_4_@rGO composites using a hydrothermal method. The 3D graphene structure mitigated nanoparticle agglomeration and volume changes, resulting in a reversible discharge capacity of 1139 mAh g^−1^ and 85% retention after 100 cycles at 400 mA g^−1^. Even at 1000 mA g^−1^, the electrode retained 665 mAh g^−1^ after 200 cycles.

Porosity has also proven crucial in enhancing performance by facilitating electrolyte diffusion and accommodating volume expansion [192]. Gao et al. [156] developed yolk–shell Fe_3_O_4_@C structures anchored on graphene, which achieved 358 mAh g^−1^ at 10 A g^−1^ and 579 mAh g^−1^ after 1800 cycles. Huang et al. [157] synthesized porous Fe_3_O_4_ NPs grown on 3D graphene foam (GF– Fe_3_O_4_), achieving 1220 mAh g^−1^ at the 500th cycle, with a linear capacity increase of ~2.2 mAh g^−1^ per cycle (Figure 17b). Furthermore, Qin et al. [158] prepared a porous Fe_3_O_4_/N-doped rGO composite using a one-step hydrothermal method (Figure 17c). Nitrogen doping improved graphene dispersion and structural adaptability, resulting in capacities of 1072.5 and 905.2 mAh g^−1^ after 100 and 250 cycles, respectively. Tian et al. [159] also synthesized a 3D Fe_3_O_4_/N-doped rGO composite, reaching reversible capacities of 1496.7 and 1184.8 mAh g^−1^ at current densities of 0.5 and 1.0 A g^−1^, respectively, after 500 cycles.

**Figure 18:**
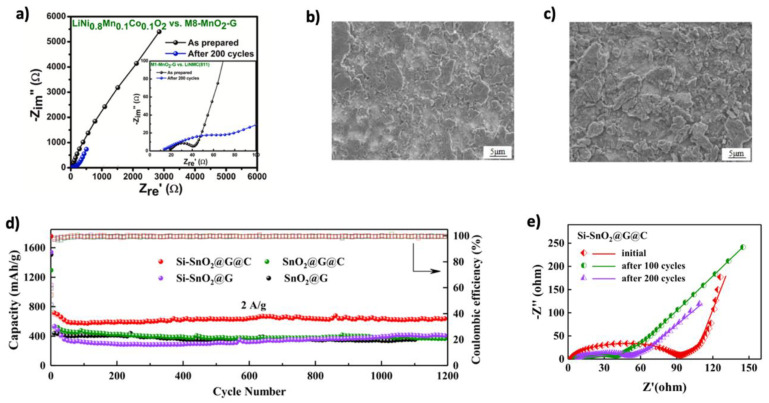
(**a**) EIS of the as-prepared full cell and after 200 cycles at 123 mA g^−1^. Adapted with permission [160]. Copyright 2022, Elsevier. (**b**,**c**) SEM images of graphene-wrapped MnCO_3_/Mn_3_O_4_ nanocomposite before and after 200 cycles, respectively. Adapted with permission [162]. Copyright 2021, Elsevier. (**d**) Cycling performance and CE at 2 A g^−1^ for 1200 cycles. (**e**) EIS of Si–SnO_2_@G@C before and after 100 and 200 cycles. Reprinted with permission [165]. Copyright 2020, American Chemical Society.

Besides iron oxides, manganese oxides (MnO_2_, Mn_2_O_3_, and Mn_3_O_4_) are attractive due to their high theoretical capacities (1232, 1019, and 936 mAh g^−1^), low voltage, and eco-friendliness [38,195]. Zaidi et al. [41], through a DFT study, explored the electrochemical behavior of MnO_2_ in LIBs, highlighting its ability to undergo phase transformations and exist in multiple oxidation states (e.g., Mn^4+^/Mn^3+^). These features facilitate redox reactions and structural adaptability during lithiation/delithiation, enhancing charge–discharge performance. When combined with graphene, MnO_2_ forms a hybrid that benefits from graphene’s high electrical conductivity, which compensates for MnO_2_’s intrinsic low conductivity, improving charge transfer. The redox activity of MnO_2_ further supports efficient lithium-ion intercalation. Wu and Fan [42] theoretically demonstrated that the MnO_2_/graphene hybrid increases lithium adsorption energy and reduces diffusion barriers, promoting faster ion transport. Graphene’s mechanical strength also helps buffer MnO_2_’s volume changes during cycling, improving structural stability. Experimentally, Ette et al. [160] created mesoporous MnO_2_ with 3D graphene (M8-MnO_2_-G), which achieved 1494 mAh g^−1^ initially and retained 1100 mAh g^−1^ after 150 cycles. EIS analysis (Figure 18a) showed minimal resistance increase after 200 cycles. Xu et al. [161] prepared MnO_2_/Graphene Films via electrophoretic deposition and annealing, forming hierarchical porous networks. The composite showed high performance: 1652.2 mAh g^−1^ at 0.1 A g^−1^ after 200 cycles and 616.8 mAh g^−1^ at 4 A g^−1^. Chen et al. [162] produced MnCO_3_/Mn_3_O_4_ NPs uniformly coated with graphene. The ternary composite demonstrated a high initial charge capacity (2457.4 mAh g^−1^), with 93.3% retention after 200 cycles at 500 mA g^−1^. SEM images (Figure 18b,c) revealed how the graphene coating suppressed aggregation and maintained structural integrity.

Among metal oxide materials, SnO_2_ is a promising alternative anode for LIBs due to its high theoretical capacity of 1494 mAh g^−1^, environmental friendliness, and low cost [196]. However, its large volume change (~300%) during cycling causes cracking and degradation, which can be mitigated by incorporating graphene for enhanced stability and conductivity [197]. Graphene integration enhances structural resilience and electrical conductivity. Liu et al. [163] synthesized SnO_2_/graphene composites using a microwave hydrothermal method, achieving discharge capacities of 1359, 1228, 1090, and 1005 mAh g^−1^ at current densities of 100, 300, 500, and 700 mA g^−1^ after 100 cycles, respectively. Further improvements were achieved using structural engineering. Liu et al. [164] prepared SnO_2_supported on orderly stacked reduced graphene oxide sheets, enhancing capacity (1080 mAh g^−1^ after 500 cycles) and stability. Duhan et al. [198] through ab initio simulations, showed that Si-doping in γ-graphyne improves lithium diffusion and stabilizes graphene. Ao et al. [165] applied this concept by creating Si–doped nO_2_ nanorods/rGO coated with C composites (Si–SnO_2_@G@C). The C shell prevented Sn aggregation and electrolyte contact, while silicon doping improved performance. The electrode achieved 654 mAh g^−1^ at 2 A g^−1^ after 1200 cycles (Figure 18d), and EIS data (Figure 18e) confirmed reduced impedance and enhanced stability. Yin et al. [199] developed a SnO/SnO_2_@graphene heterojunction composite, where DFT and experimental results revealed enhanced lithium diffusion due to the Sn–O–C interface and improved electronic conductivity. The composite retained 498.7 mAh g^−1^ at 1 A g^−11^ after 400 cycles. Gao et al. [166] introduced SnO_2_@MOF/graphene composites, using MOFs to prevent pulverization, reduce SEI instability, and maintain structural integrity. The electrode exhibited 450 mAh g^−1^ after 1000 cycles at 1 A g^−1^, outperforming bare SnO_2_.

Despite their potential, TMO–graphene composites still face challenges such as volume expansion and limited conductivity. Artificial SEI layer engineering on TMOs could enhance cycling stability by preventing structural degradation. Additionally, integrating TMOs and graphene into self-healing polymer matrices offers a promising route to autonomously repair damage from volume fluctuations, thereby extending anode durability.

In addition, various metal oxide materials have been extensively combined with graphene in composite structures to enhance electrochemical performance, driven by the growing demand in several fields—particularly energy storage. For example, TiO_2_ [167], cobalt oxides (CoO_x_) [168,200], and nickel oxide (NiO) [169], demonstrate very high specific capacities.

##### Metal Sulfide–Graphene Composites

**Figure 19:**
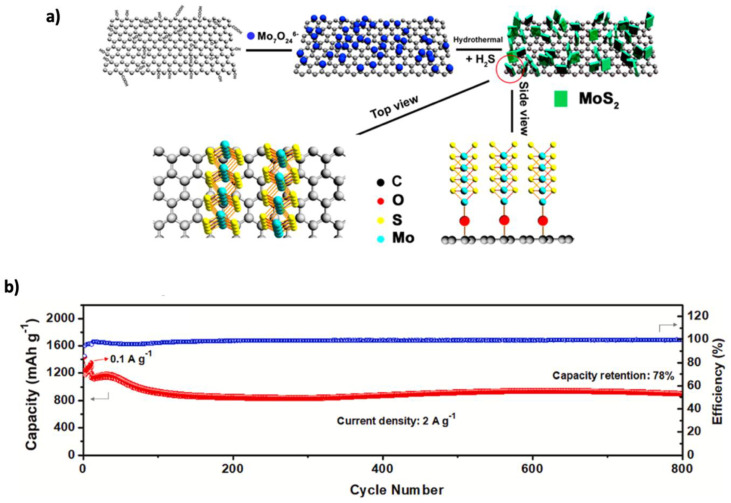
(**a**) Schematic illustration of the synthesis procedure of MoS_2_/G. Reprinted with permission [170]. Copyright 2016, American Chemical Society. (**b**) Cycling performance of FL-MoS_2_/N-G at 2 A g^−1^. Adapted with permission [172]. Copyright 2023, Elsevier.

Metal sulfides have recently gained attention as promising anode materials for LIBs due to their superior electrochemical activity, higher conductivity, and better mechanical and thermal stability compared to metal oxides. When combined with graphene, these advantages are further enhanced, as the graphene matrix improves Li^+^ adsorption, boosts electronic conductivity, and provides mechanical support that mitigates volume changes during cycling [201,202]. MoS_2_, a promising anode material for LIBs, benefits from graphene’s high surface area, mechanical strength, electrochemical properties, and lithium-ion insertion/desorption abilities. MoS_2_ also has a low toxicity and a high theoretical capacity of 670 mAh g^−1^ [10,203]. Shao et al. [204] conducted DFT calculations to investigate lithium intercalation in graphene/MoS_2_ composites, revealing that graphene enhances lithium-ion diffusion by supporting MoS_2_ layers. Their study showed that the composites exhibit high lithium storage capacity, with binding energies per lithium atom increasing as more lithium is intercalated, suggesting efficient storage for battery applications. Experimentally, Teng et al. [170] validated this by growing MoS_2_ nanosheets vertically on graphene (MoS_2_/G) (Figure 19a), which exposed more active sites and facilitated ion transport. Their composite achieved a high reversible capacity of 1077 mAh g^−1^ at 100 mA g^−1^, and still maintained 907 mAh g^−1^ at 1000 mA g^−1^ after 400 cycles. Further enhancement was reported by Xiao et al. [171] who integrated few-layered MoS_2_ with N-doped graphene ribbons (N-GRs/MoS_2_), achieving 1151 mAh g^−1^ and excellent cycling stability (92.6% retention after 600 cycles). Similarly, Liu et al. [172] synthesized flower-like MoS_2_ nanosheets encapsulated in N-doped graphene (FL-MoS_2_/N-G), resulting in improved structural integrity, better charge transfer, and reduced diffusion resistance. First principles simulations predicted more lithiation sites, and the composite delivered 1202 mAh g^−1^ at 0.2 A g^−1^ with 78% retention after 800 cycles (Figure 19b).

**Figure 20:**
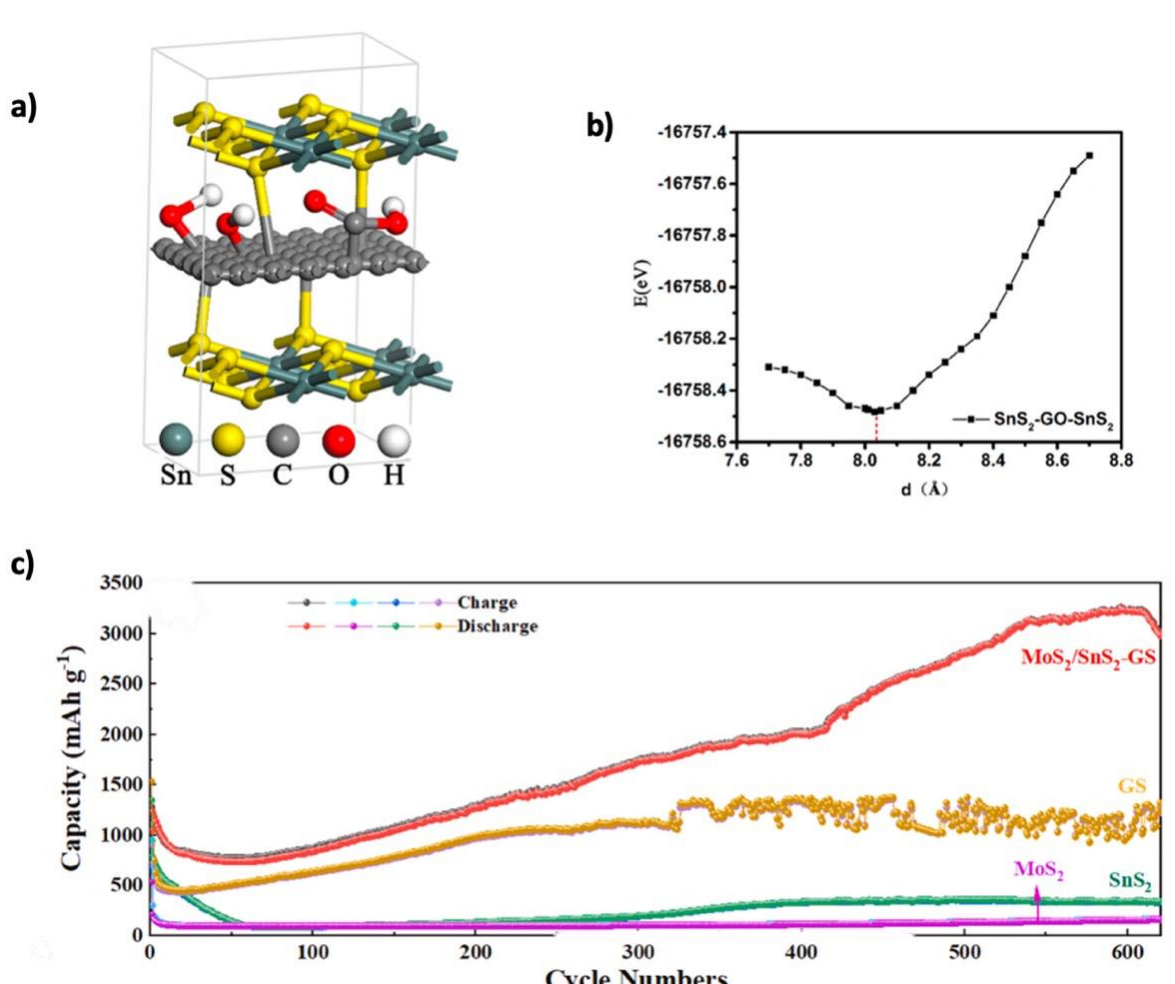
(**a**) Molecular model of the sandwich-structured SnS_2_/rGO/SnS_2_. (**b**) System energy as a function of interlayer spacing. Reprinted with permission [173]. Copyright 2019, American Chemical Society. (**c**) Cycling performance of SnS_2_, MoS_2_, GS, and MoS_2_/SnS_2_-GS at 0.5 A g^−1^ for 600 cycles. Adapted with permission [176]. Copyright 2022, Elsevier.

SnS_2_ is another notable metal sulfide with a high theoretical capacity of 1232 mAh g^−1^ and advantages such as abundance and non-toxicity [8,37,205]. However, it suffers from volume expansion and low conductivity. These limitations are effectively addressed by hybridizing SnS_2_ with graphene [205]. Jiang et al. [173] fabricated a SnS_2_/rGO/SnS_2_ hybrid composite, consisting of few-layered SnS_2_ nanosheets covalently decorated on both sides of rGO, as shown in Figure 20a, using a simple hydrothermal method. The composite’s sandwich-like structure, with an interlayer spacing of ∼8.03 Å (Figure 20b), allows fast ion transport and prevents SnS_2_ nanosheet stacking. This structure led to impressive electrochemical performance, with a reversible capacity of 1357 mAh g^−1^ at 0.1 A g^−1^ after 200 cycles, 96.6% capacity retention, and a rate performance of 844 mAh g^−1^ at 10 A g^−1^. Theoretical studies by Idisi et al. [206] support these findings, showing that SnS_2_/graphene/SnS_2_ heterostructures possess low cohesive energies and strong electronic coupling, with unoccupied states in the DOS states suggesting efficient charge transfer and high performance. He et al. [174] synthesized SnS_2_-rGO microspheres with S-doped graphene, enhancing interfacial contact and lithium reactivity. This resulted in capacities of 1647.8 mAh g^−1^ at 0.1 A g^−1^ and 1177.2 mAh g^−1^ at 1 A g^−1^ after 400 cycles. A more complex design was introduced by Sheng et al. [176], who fabricated a three-layered MoS_2_/SnS_2_–S-doped graphene anode (MoS_2_/SnS_2_-GS). This heterostructure provided enhanced surface kinetics and lithium adsorption, achieving outstanding capacities of 2007.4 mAh g^−1^ at 0.1 A g^−1^ and 3224.3 mAh g^−1^ at 0.5 A g^−1^ after 600 cycles. Figure 20c illustrates how the design overcomes issues such as electrode degradation due to stacking and volume fluctuations in MoS_2_ and SnS_2_. To further improve performance, Gao et al. [175] introduced a 3-aminophenol (AP) linker in SnS_2_/N-doped graphene. The AP molecule enhanced binding energies, controlled SnS_2_ growth, and provided additional lithium storage sites, achieving 1101.3 mAh g^−1^ with minimal capacity fading (0.04% per cycle over 200 cycles). Finally, Yin et al. [177] developed a 3D Mo-doped SnS_2_/SnO_2_ composite integrated with N-doped graphene. The Mo doping (Mo^6+^ vs. Sn^4+^) modified the electronic structure and shortened Li^+^ transfer paths. This composite exhibited a capacity of 2052.4 mAh g^−1^ after 100 cycles at 0.1 A g^−1^.

Metal sulfide–graphene composites offer significant advantages over TMO-based systems, especially in terms of capacity, rate capability, and cycling stability. The high theoretical capacities of MoS_2_ and SnS_2_, combined with the conductivity and structural support of graphene, make these hybrids highly promising. Nonetheless, long-term stability issues like structural degradation still pose challenges. Addressing these requires advanced approaches such as heterostructure engineering, doping strategies, and interfacial optimization. While the performance is impressive, further development is essential to match the energy density and scalability of more mature electrode materials.

Moreover, many researchers have shown interest in studying various metal sulfide materials (e.g., manganese sulfide (MnS) [178,207], cobalt sulfides (CoS_x_) [179,208], nickel sulfides (NiS_x_) [180], etc.), which also possess high theoretical capacities and several advantages that make them a focus of interest.

## 4. Conclusions and Future Prospects

DFT has proven to be an essential tool for exploring the electronic and structural properties of graphene-based electrodes in lithium-ion batteries. While its effectiveness is evident in revealing atomic-level phenomena, current implementations face notable challenges especially when dealing with complex systems involving defects, interfaces, and lithium diffusion. To overcome these limitations, future research should continue to refine DFT-based methods by adopting advanced exchange-correlation functionals such as hybrid and meta-GGA types and incorporating corrections like DFT+U to better capture electron localization effects. TDDFT and multi-scale modeling approaches also offer promising routes for describing dynamic processes more accurately. Moreover, integrating machine learning with DFT can accelerate materials discovery and enhance predictive accuracy. Bridging computational insights with experimental validation will be essential to translate theoretical advancements into practical improvements in electrode design, contributing to the development of next-generation energy storage systems.

## Figures and Tables

**Table 1:** Comparison between theoretical predictions and experimental results for various graphene-based anode materials used in lithium-ion batteries. * and ** indicate that values and references are derived from different sources within the same row.

Graphene Modification	Experimental Capacity (mAh g^−1^)	Theoretical Capacity (mAh g^−1^)	Li^+^ Adsorption (eV)	Li^+^ Diffusion (eV)	Ref.
Porous graphene	910 *	2857.7 **	1.80 **	0.37–0.39 **	[60,61] *^,^ **
Defective graphene	210 *	1675 **	0.028 to 0.052 **	_____	[83,84] *^,^ **
B-doped graphene	1549 *	2271 **	0.759 **	0.454 **	[85,86] *^,^ **
N-doped graphene	1043 *	1262 **	1.26–1.19	_____	[85,87] *^,^ **
Si/graphene	2753 *	2896 **	−3.80 **	_____	[29,88] **^,^ *
MoO_2_/graphene	1037 *	1411 **	−0.87 **	0.077 **	[89,90] *^,^ **
WS_2_/graphene	421 *	588.16 **	−2.33 to −1.99 **	0.24−0.28 **	[51,91] *^,^ **
SnS_2_/graphene	766.3 *	1330 **	1.97 to 3.22 **	0.21 **	[52,92] **, *

**Table 2:** Comparison of key anode materials for LIBs, highlighting their theoretical capacities, advantages, and major limitations.

Material	Theoretical Capacity (mAh g^−1^)	Main Limitations	Key Advantages
Graphene	~744	Moderate storage capacity as a pure material	High electrical conductivity, large surface area, mechanical flexibility, stable SEI formation, and long cycle life
Si	~4200	Severe volume expansion (~300%), unstable SEI formation, and rapid capacity fading	Extremely high theoretical capacity, earth abundance, low cost, environmentally friendly, and high melting points suitable for thermal stability
Transition Metal Oxides	~1000–1200 (e.g., Fe_3_O_4_, MnO_2_)	Significant volume expansion, poor electrical conductivity, and low cycling stability	High theoretical capacities, natural abundance, non-toxicity, corrosion resistance, and conversion-type lithiation mechanism
Metal Sulfides	~600–1200 (e.g., MoS_2_, SnS_2_)	Poor cycling stability and slow Li^+^ diffusion kinetics	Layered structure for Li^+^ intercalation, moderate to high capacities, and tunable electrochemical properties

**Table 3:** Summary of graphene anodes and their electrochemical parameters for LIBs.

Materials	Reversible Capacity (mAh g^−1^) at Current Density (A g^−1^)	Cycling Performance	Rate Performance	Ref.
			Capacity (mAh g^−1^) at Current Density (A g^−1^)	
Pristine Graphene				
GNSs	540 @ 0.05	290 mAh g^−1^ after 20 cycles	**______**	[136]
GNSs+CNTs	730 @ 0.05	480 mAh g^−1^ after 20 cycles	**______**	[136]
GNSs+C_60_	784 @ 0.05	600 mAh g^−1^ after 20 cycles	**______**	[136]
GNSs	600 @ 74.5	500 mAh g^−1^ after 50 cycles	**______**	[137]
GNS-I	407 @ 0.1	269 mAh g^−1^ after 100 cycles	**______**	[138]
GNS-II	723 @ 0.1	400 mAh g^−1^ after 100 cycles	**______**	[138]
GNS-III	1348 @ 0.1	691 mAh g^−1^ after 100 cycles	**______**	[138]
Doped Graphene				
N-doped graphene	1040 @ 0.05	872 mAh g^−1^ after 30 cycles	199 @ 25	[85]
B-doped graphene	1549 @ 0.05	1227 mAh g^−1^ after 30 cycles	235 @ 25	[85]
N-doped Porous Graphene Hybrid Nanosheets	1971.2 @ 0.1	90.38% after 1000 cycles	374.2 @ 2	[139]
N-doped graphene	950 @ 0.1	>500 mAh g^−1^ after 150 cycles	150 @ 5	[140]
N-doped carbon/rGO	1100 @ 0.1	535 mAh g^−1^ after 1200 cycles	45 @ 20	[141]
N-doped carbon graphene framework	2018 @ 0.5	93% after 10,000 cycles	340 @ 40	[142]
Sulfur-doped graphene nanosheets (S-GNSs)	870 @ 0.374	290 mAh g^−1^ after 500 cycles	285 @ 11.16	[143]
Si-doped graphene	**______**	86% after 400 cycles	~100 @ 4	[144]
3D crumpled B- and N-co-doped graphene nanosheets (NBGs-1000)	909 @ 0.05	877 mAh g^−1^ after 125 cycles	318 @ 2	[145]
N and S co-doped graphene	1016 @ 0.1	788.2 mAh g^−1^ after 50 cycles	250.1 @ 20	[146]
S/N co-doped aerogels (SNGA-II)	981.4 @ 0.1	1109.8 mAh g^−1^ after 400 cycles	~350 @ 0.8	[147]
Si-N co-doped graphene	644 @ 0.1	578.75 mAh g^−1^ after 500 cycles	240 @ 5	[148]
Graphene-Based Composites				
graphene/Si nanocomposites (SGE)	2753 @ 0.3	800 mAh g^−1^ after 30 cycles	**______**	[88]
3D Mesoporous Si@graphene	1480 @ 0.1	89.1% after 200 cycles	659 @ 10	[149]
Si@graphene	**______**	1063.2 mAh g^−1^ after 100 cycles	1360.9 @ 3	[150]
Sandwich-graphene/Si	1575.5 @ 0.1	1085.6 mAh g^−1^ after 500 cycles	258.4 @ 5	[151]
Si@N-doped Graphene Cages	2350 @ 0.1	900 mAh g^−1^ after 200 cycles	890 @ 5	[152]
Si/N-doped Graphene	1533 @ 0.2	97% after 50 cycles	200 @ 10	[153]
Sandwich Fe_3_O_4_/Graphene film	896 @ 2	798 mAh g^−1^ after 300 cycles	598 @ 20	[154]
3D Fe_3_O_4_@rGO	~1000 @ 0.4	1139 mAh g^−1^ after 100 cycles	786 @ 3.2	[155]
Yolk–shell Fe_3_O_4_@C	1143 @ 0.1	579 mAh g^−1^ after 1800 cycles	358 @ 10.0	[156]
3D graphene foam (GF– Fe_3_O_4_)	**______**	~1220 mAh g^−1^ after 500 cycles	~500 @ 5	[157]
porous Fe_3_O_4_/N-rGO	1094.9 @ 0.1C	905.2 mAh g^−1^ After 250 cycles	884.7 @ 1C	[158]
3D Fe_3_O_4_/N-doped rGO	**______**	1184.8 mAh g^−1^ after 500 cycles	455.1 @ 2	[159]
Mesoporous MnO_2_/3D graphene	1512 @ 0.24	1496.7 mAh g^−1^ after 500 cycles	780 @ 12.3	[160]
MnO_2_/Graphene Films	~800 @ 0.1	1652.2 mAh g^−1^ after 200 cycles	616.8 @ 4	[161]
Graphene-wrapped MnCO_3_/Mn_3_O_4_	~1300 @ 0.5	1522.8 mAh g^−1^ after 200 cycles	605.5 @ 5	[162]
SnO_2_/graphene	~800 @ 1	677 mAh g^−1^ after 1000 cycles	790 @ 1	[163]
SnO_2_ /stacked graphene	~366 @ −	1080 mAh g^−1^ after 500 cycles	~400 @ 5	[164]
Si–SnO₂ nanorods/rGO @ C	1127 @ 0.1	654 mAh g^−1^ after 1200 cycles	~600 @ 5	[165]
SnO_2_@ metal–organic framework (MOF)/graphene	**______**	450 mAh g^−1^ after 1000 cycles	324 @ 2	[166]
3D TiO_2_-graphene	271 @ 0.017	264 mAh g^−1^ after 500 cycles	158 @ 1.7	[167]
Co_3_O_4_ nanowall@graphene	~800 @ 0.1	>600 mAh g^−1^ after 500 cycles	**______**	[168]
NiO@graphene	886 @ 0.05	205 mAh g^−1^ after 500 cycles	742 @ 5	[169]
MoS_2_/graphene	1077 @ 0.1	907 mAh g^−1^ after 400 cycles	890 @ 1	[170]
N-doped graphene ribbons/MoS_2_	1151 @ 0.1	92.6% after 600 cycles	499.3 @ 8	[171]
Flower-like MoS_2_/N-doped graphene	1202 @ 0.2	78% after 800 cycles	835 @ 5	[172]
SnS_2_/rGO/SnS_2_	1295 @ 0.1	909 mAh g^−1^ after 400 cycles	844 @ 10	[173]
SnS_2_/S-doped rGO	1630.9 @ 0.1	1177.2 mAh g^−1^ after 400 cycles	1050.0 @ 2	[174]
SnS_2_/N-doped graphene	1101.3 @ 0.1	fading of 0.04% per cycle for 200 cycles	656.3 @ 2	[175]
Three-layer MoS_2_/SnS_2_ on S-doped graphene	1100 @ 0.1	3224.3 mAh g^−1^ after 600 cycles	1062.8 @ 3	[176]
3D Mo–SnS₂/SnO₂–N-doped graphene composite	**______**	2052.4 mAh g^−1^ after 600 cycles	617.33 @ 3	[177]
MnS/rGO	800 @ 1	640 mAh g^−1^ after 400 cycles	580 @ 2	[178]
CoS_2_-quantum-dots anchored graphene GNSs	1025.5 @ 0.1	831 mAh g^−1^ after 300 cycles	411 @ 10	[179]
NiS_2_/graphene	1200 @ 0.1	810 mAh g^−1^ after 1000 cycles	740 @ 1	[180]
